# Designing Dietary Recommendations Using System Level Interactomics Analysis and Network-Based Inference

**DOI:** 10.3389/fphys.2017.00753

**Published:** 2017-09-28

**Authors:** Tingting Zheng, Yueqiong Ni, Jun Li, Billy K. C. Chow, Gianni Panagiotou

**Affiliations:** ^1^Systems Biology and Bioinformatics Group, Faculty of Sciences, School of Biological Sciences, The University of HongKong, Hong Kong, Hong Kong; ^2^Faculty of Science, School of Biological Sciences, The University of Hong Kong, Hong Kong, Hong Kong; ^3^Department of Systems Biology and Bioinformatics, Leibniz Institute for Natural Product Research and Infection Biology, Hans Knöll Institute, Jena, Germany

**Keywords:** gene expression, enrichment score, diet-disease associations, diseases, protein-protein Interaction network

## Abstract

**Background:** A range of computational methods that rely on the analysis of genome-wide expression datasets have been developed and successfully used for drug repositioning. The success of these methods is based on the hypothesis that introducing a factor (in this case, a drug molecule) that could reverse the disease gene expression signature will lead to a therapeutic effect. However, it has also been shown that globally reversing the disease expression signature is not a prerequisite for drug activity. On the other hand, the basic idea of significant anti-correlation in expression profiles could have great value for establishing diet-disease associations and could provide new insights into the role of dietary interventions in disease.

**Methods:** We performed an integrated analysis of publicly available gene expression profiles for foods, diseases and drugs, by calculating pairwise similarity scores for diet and disease gene expression signatures and characterizing their topological features in protein-protein interaction networks.

**Results:** We identified 485 diet-disease pairs where diet could positively influence disease development and 472 pairs where specific diets should be avoided in a disease state. Multiple evidence suggests that orange, whey and coconut fat could be beneficial for psoriasis, lung adenocarcinoma and macular degeneration, respectively. On the other hand, fructose-rich diet should be restricted in patients with chronic intermittent hypoxia and ovarian cancer. Since humans normally do not consume foods in isolation, we also applied different algorithms to predict synergism; as a result, 58 food pairs were predicted. Interestingly, the diets identified as anti-correlated with diseases showed a topological proximity to the disease proteins similar to that of the corresponding drugs.

**Conclusions:** In conclusion, we provide a computational framework for establishing diet-disease associations and additional information on the role of diet in disease development. Due to the complexity of analyzing the food composition and eating patterns of individuals our *in silico* analysis, using large-scale gene expression datasets and network-based topological features, may serve as a proof-of-concept in nutritional systems biology for identifying diet-disease relationships and subsequently designing dietary recommendations.

## Introduction

Diet plays a very important role in maintaining health and preventing diseases by influencing the physiological state of humans in a number of ways. The antioxidant properties of molecules in fruits and vegetables are known to be protective against free radical damage (Moo-Huchin et al., 2015; Vinha et al., 2015). Especially savoy cabbage, spinach and collard were proved to exhibit high antioxidant potential (>80%) (Vinha et al., 2015). There has been evidence of the metabolic effects of diet on aging and age-related diseases (Mattison et al., 2000; Speakman and Mitchell, 2011; Johnson et al., 2013; Solon-Biet et al., 2014; Finkel, 2015). It has been shown that reducing food intake without malnutrition can prolong lifespan (Mattison et al., 2000; Lin et al., 2002), whereas metabolic sensors regulated by food nutrients, such as mechanistic target of rapamycin (mTOR), sirtuins and adenosine monophosphate (AMP)-activated protein kinase (AMPK), may contribute to the aging phenotype (Finkel, 2015). On the other hand, food may be a risk factor for certain diseases, including the development of cancer by affecting processes such as cell differentiation and apoptosis, as well as the hormonal regulation of cellular functions (Mayne et al., 2016). For instance, red meat and alcohol have been associated with colorectal cancer and cancers of the gastrointestinal tract, respectively (Wiseman, 2008). High-fat diary foods have been found to contribute to the risk of Alzheimer's disease (Knight et al., 2014). In addition, as a lifestyle factor, diet can act on the epigenome and alter relevant gene expression profiles, potentially influencing the pathogenesis of type 2 diabetes mellitus (Barres and Zierath, 2016). Since food composition and eating patterns are highly complex, identifying relationships between diet and diseases remains a challenging task. New approaches are needed to identify the role of different foods in disease development and to design dietary recommendation.

Systems biology may serve as a solution here and it has been considered as a powerful tool for integrating multi-omics data to perform extensive analyses and to gain a global understanding of how diet contributes to health and disease (Panagiotou and Nielsen, 2009; Badimon et al., 2016). Using systematic approaches, we previously identified plant-based food-disease and food-drug associations through interactions between food bioactive compounds and disease proteins (or drug targets) (Jensen et al., 2014, 2015a). We proposed a statistical framework for screening specific phytochemicals that perturb drug targets and disease-related pathways (Jensen et al., 2014), and pinpointed key proteins in colon cancer that may be perturbed by dietary interventions (Westergaard et al., 2014). Investigating food-drug associations led to the recognition of foods that may negatively interfere with drug treatment for specific diseases, providing a platform for designing dietary recommendations in association with certain medications (Jensen et al., 2015a). We also developed a database linking foods with their small-molecule components (http://www.cbs.dtu.dk/services/NutriChem-1.0/), which provides a comprehensive source for chemoinformatics-based inference of food-disease interactions (Jensen et al., 2015b). Moreover, diet has not only a direct effect on the host but also an indirect effect on gut microbiota alterations (David et al., 2014), which may contribute to pathogenesis of disorders such as inflammatory bowel diseases (Albenberg and Wu, 2014). In a previous study of our group (Ni et al., 2015), we investigated at molecular level how diet could affect gut microbiota functionality and revealed that small-molecule nutrients mainly affected the expression of bacterial genes related to metabolic pathways, providing useful insights for microbiome-targeted dietary recommendations. The high-fiber, low-fat diet can result in remarkable changes in the composition and functions of gut microbiota, especially the ones related to butyrogenesis and secondary bile acid synthesis, which are aspects known to affect cancer risk (O'Keefe et al., 2015). However, the studies described above mainly relied on known dietary chemical compositions and their direct interactions with disease proteins, microbiome proteins or drug targets, ignoring the global effect that diet may induce at the gene expression level and possible associations with disease gene expression signatures.

Based on the hypothesis that if a disease state is signified by a specific set of genome-wide expression changes, then introducing a factor that could reverse the disease gene expression signature will naturally lead to a therapeutic effect (Sirota et al., 2011), a range of methods have been developed and successfully used for drug repositioning that involves screening of new clinical applications of well-known and approved drugs (Lamb et al., 2006; Iorio et al., 2010; Shigemizu et al., 2012; van Noort et al., 2014). Among numerous application examples of this approach, Lamb et al. (2006) recognized that 4,5-dianilinophthalimide (DAPH) might be a potential therapeutic agent for Alzheimer's disease (AD) and that sirolimus could be tested in acute lymphoblastic leukemia (ALL) patients with dexamethasone resistance. Subsequently, Pacini et al. developed DvD, which provides a pipeline for comparisons between drug and disease gene expression profiles (Pacini et al., 2013). Another example is the identification of 247 drugs that consistently significantly reversed lung cancer gene expression changes (Fortney et al., 2015), from which spiperone and pimozide had been reported as promising drugs for lung cancer. Using systems-level approaches, our objectives here are: (1) to establish diet-disease correlations and identify diets that could either reverse or induce the expression of disease-related genes, by calculating pairwise similarity scores between diet and disease gene expression signatures; (2) to understand mechanistic similarities and differences in the mode of action of diet- and drug-based interventions, though integration of gene expression signatures, advanced topological analysis and information on experimentally validated associations of diet with disease phenotypes; (3) to predict synergistic food pairs, since humans do not normally consume foods in isolation, that may facilitate the design of more complete dietary recommendations for specific groups at risk by applying different algorithms.

## Materials and methods

### Diet, drug, and disease gene expression data

For diet, drugs and diseases, only the datasets that have well-controlled experimental design including gene expression profiles measured before and after interventions (here referring to diet, disease or drug) under the same conditions were used for analysis. In addition, we only kept the datasets using oligonucleotide microarrays and the ones with available annotations to Entrez gene IDs. The raw and processed gene expression profiles of foods and diseases were downloaded from NCBI GEO (Edgar et al., 2002) and ArrayExpress (Parkinson et al., 2005). We processed gene expression profiles of 18 foods from 23 datasets (Table S1) and 111 diseases from 212 datasets (Table S2). Using the classification proposed by Goh et al. (2007), 74 of the 111 diseases were further classified into 17 disease classes. The gene expression datasets for 121 drugs corresponding to 48 diseases (Table S3) were downloaded from NCBI GEO, ArrayExpress, Cmap (Lamb et al., 2006) and DrugMatrix® (Ganter et al., 2005; https://ntp.niehs.nih.gov/drugmatrix/index.html).

In terms of data processing, RMA normalization (Irizarry et al., 2003) was applied to raw data from the Affymetrix microarray platforms, including the Affymetrix Human Genome U133 Plus 2.0 Array, Affymetrix Human Genome U133A Array and Affymetrix Human Gene 1.0 ST Array. For other microarray platforms, processed data were used. Probe sets were mapped to Entrez genes based on the annotations provided by GEO. Multiple probe sets mapped to the same gene were converted to the average intensities. Probe sets mapped to multiple gene identifiers or with missing values in over 20% samples were removed.

To increase the data size, datasets from rats and mice were also included (Iskar et al., 2013). In processing these datasets, we only retained genes with human orthologs based on NCBI Homologene (Sayers et al., 2009), and the orthologous genes were used for subsequent analyses. Previous studies have shown that particular transcriptional changes induced by different growth conditions, stress or disease can be conserved between species (Miller et al., 2010; Zheng-Bradley et al., 2010; Dowell, 2011), so it is feasible to combine datasets from rats, mice and human to generate unique gene expression signature lists.

### Enrichment score (ES) calculation

**Figure 1:**
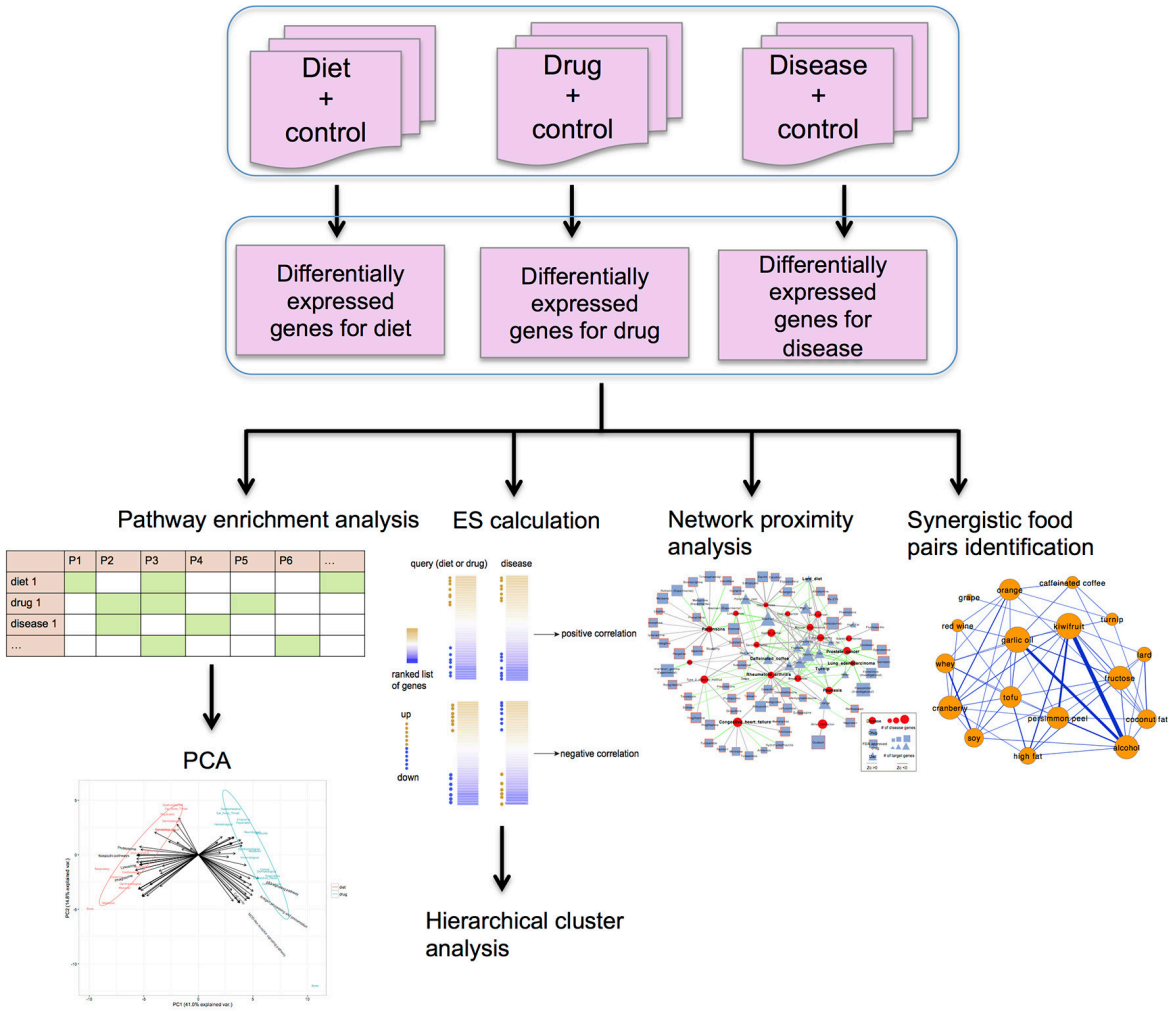
The framework of our methodology applied in this study. The differentially expressed (DE) genes of diets, drugs and diseases were detected using the R package Rankprod, by comparison with respective control groups. The R package Gostats was applied for KEGG pathway enrichment analysis. The identified significant pathways for diets, drugs and diseases were used to construct the score matrix for the PCA. Diet-disease and drug-disease associations were calculated using the enrichment score (ES) method in DvD package. Hierarchical analysis was performed to show disease similarity based on the ES scores. Using a python script developed by Guney et al. (2016), we measured the proximity values between DE genes of diets or drugs and diseases within a human protein-protein interaction (PPI) network. Synergistic food pairs were identified using the approaches from Bansal et al. (2014).

The R package RankProd (Hong et al., 2006), which is based on the rank product method (Breitling et al., 2004) that could overcome the platform heterogeneity, was used to detect differentially expressed (DE) genes by integrating various experimental datasets (Figure 1). With a false discovery rate (FDR) ≤0.05, significantly DE genes for each food/drug/disease were identified.

The ES method developed by Pacini et al. (2013) was applied to establish diet-disease and drug-disease associations. Genes were rank-ordered based on the log fold change calculated by RankProd. The number of genes used for ES calculation was determined dynamically by the number of significantly DE genes. Afterwards, the enrichment scores were calculated using the Kolmogorov-Smirnov-based statistic (Subramanian et al., 2005; Iorio et al., 2013) for each food/drug gene set against each disease gene set. If the FDR-corrected *P*-value was less than 0.05, then diet-disease pairs (or drug-disease pairs) were considered to be significantly associated. Furthermore, a positive enrichment score indicated correlated diet-disease pair (or drug-disease pair), whereas a negative enrichment score indicated anti-correlated relationship.

### Hierarchical cluster analysis

Hierarchical clustering was used to show disease similarity based on the enrichment scores of diseases across all the drugs here. After extracting the enrichment score between each disease and each drug, the R package Pvclust (Suzuki and Shimodaira, 2006) was employed to compute Pearson correlation coefficients as the distance metric between disease pairs and to perform hierarchical clustering.

### Principal component analysis (PCA) at the pathway level

KEGG pathway enrichment analysis was performed for each diet and drug using the R package Gostats (Falcon and Gentleman, 2007), with significantly enriched pathways identified at FDR < 0.05. Then the diet-disease and drug-disease pairs with negative enrichment scores (anti-correlations) were retrieved and grouped into disease classes as described above. For each disease class, we first constructed a binary matrix: if the pathway was enriched for one food or drug then it was marked as 1, otherwise as 0. Subsequently, for each disease class we aggregated the pathways enriched for all foods or drugs into “diet” and “drug,” respectively. To avoid the bias caused by different numbers of food-disease and drug-disease pairs, we used the proportion of foods or drugs marked as “1” as the final score of each enriched pathway for each disease class. This score matrix was used for PCA.

Using the same method and cutoff, pathway enrichment analysis was also performed for disease-induced gene expression profiles. Here, the disease-related pathways for foods or drugs were defined as the common enriched pathways between gene expression signatures of diseases and foods (or drugs).

### Network proximity analysis

The proximity measure proposed by Guney et al. (2016) was used to further analyze the diet-disease and drug-disease pairs with anti-correlated relationships (negative enrichment score). For diseases with FDA-approved (or clinically investigated) drugs, the disease-associated genes were retrieved from Menche et al. (2015). In total, disease genes were retrieved for 19 diseases corresponding to 63 FDA approved (or clinically investigated) drugs and 18 anti-correlated foods. The interactome data were obtained from the PPI network compiled by Menche et al. (2015). Using the Python script located at https://github.com/emreg00/toolbox (Guney et al., 2016), the proximity measures between DE genes of foods or drugs and the disease genes were calculated.

### Chemical similarity between dietary compounds and drugs

Canonical SMILES (simplified molecular input entry system) of dietary bioactive compounds were retrieved from NutriChem (Jensen et al., 2015b) and SMILES of drugs were retrieved from DrugBank (Law et al., 2014), PubChem (Bolton et al., 2008), and BindingDB (Gilson et al., 2016). The pairwise similarity scores between dietary compounds and drugs were measured by Morgan (ECFP like) fingerprints and Tanomito coefficients. The cutoff for similarity scores was set at 0.4.

### Identifying synergistic pairs

Three approaches from Bansal et al. (2014) were adopted here: (1) the Rank 2 method, which identifies a set of core genes defined by statistically significantly DE genes in at least one food administration and uses these genes to estimate an interaction score by calculating the number of overlapping genes, taking directionality into account; (2) the Rank 4 method, which computes a Pearson correlation between gene expression profiles of two foods using DE genes in at least one food; and (3) the Rank 9 method, which uses the rank-aggregation method to combine food-pair similarity results obtained from gene expression correlations, commonly affected pathways and functions. A detailed description of these methods is available from Bansal et al. (2014). Food pairs serving as a gold standard met two criteria: (1) the food pair should share at least one same association with the same disease, and (2) the total sum of the number of supported references, as recorded in NutriChem (10 of 18 foods from our data can be found in this database) (Jensen et al., 2015b), for each shared disease association between 2 foods should be more than 10. The method that recovered most of the gold standard pairs was used for identifying synergistic food pairs.

## Results

### Evaluation of the enrichment score method for associating diet with diseases based on global gene expression signatures

We collected gene expression profiles for 111 diseases and 121 drugs indicated for at least one disease (Tables S2, S3) from public databases. Pairwise correlations between drugs and diseases were calculated using the ES method (Pacini et al., 2013), after which correlated and anti-correlated drug-disease pairs were identified (Figure 1) (see Materials and Methods for more details). The essence of this approach is to quantify the similarity of gene expression signatures between drugs and diseases by calculating the consistency of the rank of drug gene expression signatures in the ranked gene lists for diseases, and vice versa. The ranked gene lists are generated according to their differential expression (commonly ranked by fold change). If the most up-regulated (or down-regulated) genes for drug tend to be enriched at the top (or bottom) of ranked list for disease, and vice versa, it indicates similar (or correlated) gene expression profiles between drug and disease, whereas the enrichment of down-regulated (or up-regulated) genes at the top (or bottom) of the ranked list reflects anti-correlated drug-disease pair. Of 160 known drug indications for diseases, 38 were identified to be significantly anti-correlated according to the ES. This percentage of recovery is consistent with a previous study on drug repositioning (Shigemizu et al., 2012), since globally reversing the disease expression signature is not a prerequisite for a drug to be active. We next investigated the recovery of known drug and disease relationships at the disease class level (17 classes in total). Cancer was the only disease class for which we recovered significantly more known drug-disease relationships using the ES method than random guessing (Fisher's exact test *P* = 0.0004) (Table S4). Notably, cancer is considered a type of disease that can induce global changes in gene expression (Yeoh et al., 2002; Andersson et al., 2005; Sotiriou and Pusztai, 2009), suggesting that associations with the corresponding drugs may be more easily recovered.

**Figure 2:**
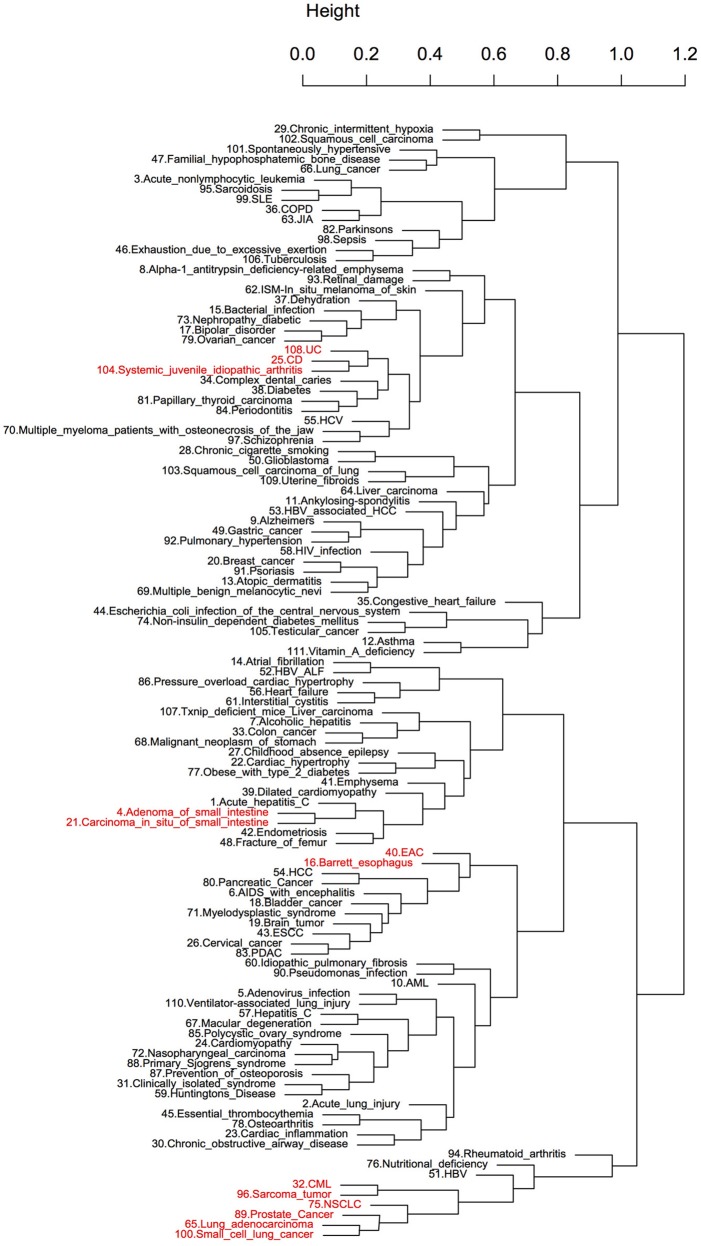
Hierarchical clustering of diseases using diet-disease enrichment scores. The number before each disease name was used as a disease ID. The clustering is based on the enrichment scores of diseases across all foods. The clusters highlighted with red are known to share characteristic pathophysiology.

To further evaluate the efficacy of the ES method, pairwise scores were calculated between the expression signatures of 18 foods (Table S1) and 111 diseases. The objective of this analysis was to evaluate whether the relatively small size of the diet-induced gene expression datasets available in public databases could reveal shared pathophysiological characteristics of the diseases. Hierarchical clustering was performed to determine whether this method could reflect disease similarity based on disease correlation profiles across all 18 foods (Figure 2). We identified several clusters of diseases with potential commonality. The most representative was a large cluster of cancers that included chronic myelogenous leukemia (CML), prostate cancer, different subtypes of lung cancer and sarcomas. Furthermore, Crohn's disease (CD) and ulcerative colitis (UC), which are collectively known as inflammatory bowel disease (IBD), as well as another inflammatory disease, systemic juvenile idiopathic arthritis, clustered together. Similar clusters were reported in a previous drug repositioning study (Sirota et al., 2011). However, more commonalities in pathophysiology were revealed here due to the larger disease dataset used. Esophageal adenocarcinoma (EAC) and Barrett's esophagus (BE), which is the main risk factor of EAC (Sikkema et al., 2010), appeared together. Adenoma of the small intestine and carcinoma *in situ* of the small intestine, which are both subtypes of cancer of the small intestine, also clustered together. Most of the clusters identified above were also retrieved when the hierarchical clustering was based on correlation profiles of each disease across all the drugs (121) used in our study (Figure S1).

### Positive and negative diet-disease correlation profiles based on global gene expression signatures

**Figure 3:**
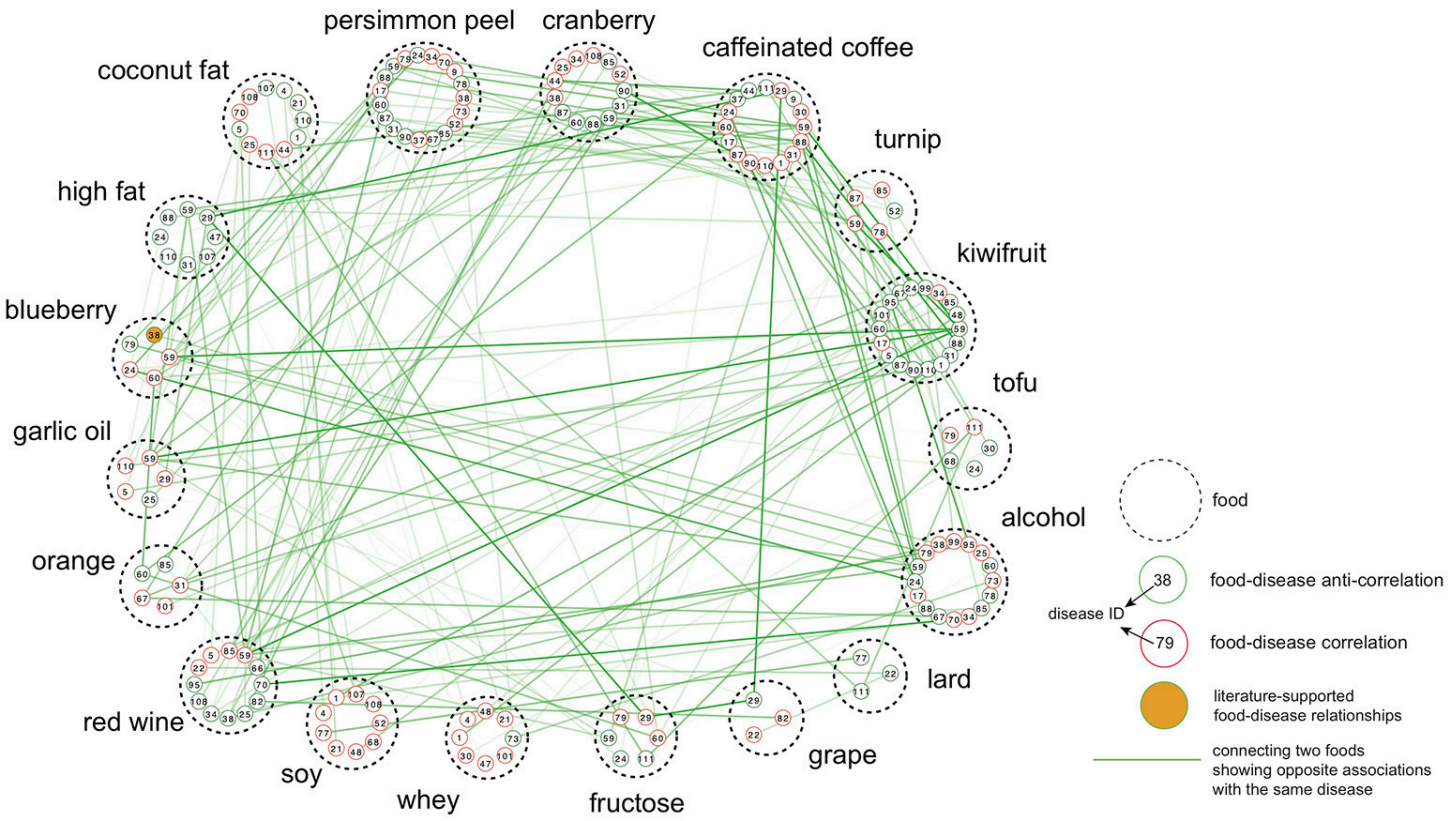
Food-centric diet-disease network. Each node is a disease associated with the food represented by dashed circle. The number inside each node is the disease ID also used in Figure 2. Only associations with absolute enrichment score values >0.13 were selected for visualization. Nodes are connected if two foods show opposite associations toward the same disease, and the edge thickness is proportional to the difference between two enrichment scores.

Having illustrated that the ES method was able to retrieve known drug therapeutic indications and pathophysiological characteristics, this method was applied to identify diets that could be recommended or avoided in a particular disease state. With an FDR-corrected *P* < 0.05 (1,000 permutations), we identified 485 diet-disease pairs that showed an anti-correlation relationship, indicating that diet might have a positive impact against disease development. We also retrieved 472 pairs that showed correlation relationships, implying that these foods should be avoided in a disease state (Table S5). Interestingly, no foods had a solely positive or negative correlation with the entire range of diseases, indicating that there are probably no “generally good” or “generally bad” foods (Figure 3). The network shown in Figure 3 also reveals that, even in cases of anti-correlation between a food and a disease, consuming that food simultaneously with another food may compromise its beneficial effect. For example, kiwifruit had an enrichment score = −0.451 against Huntington's disease, whereas blueberry had an enrichment score = 0.135.

The top-ranked foods in the list of most anti-correlation relationships were kiwifruit (44) and tofu (37). Kiwifruit was anti-correlated with cardiomyopathy (ES = −0.154) and several cancers, such as cervical cancer (ES = −0.056) and lung cancer (ES = −0.089), among other diseases. Tofu (bean curd) showed anti-correlated relationships with breast cancer (ES = −0.104) and cardiomyopathy (ES = −0.152). Another soy product, soy protein isolate, was also anti-correlated with bladder cancer (disease ID: 18, ES = −0.099), breast cancer (disease ID: 20, ES = −0.112), ovarian cancer (disease ID: 79, ES = −0.083) and prostate cancer (disease ID: 89, ES = −0.220). Persimmon peel was anti-correlated with diseases belonging to different classes, such as pancreatic ductal adenocarcinoma (PDAC, disease ID: 83, ES = −0.118), cardiomyopathy (disease ID: 24, ES = −0.285), Huntington's disease (disease ID: 59, ES = −0.237) and rheumatoid arthritis (RA, disease ID: 94, ES = −0.045). For the cancer disease class in particular, no foods were found to have solely anti-correlations with the entire range of cancers. Tofu showed the highest number of anti-correlated relationships with cancer diseases (21), followed by alcohol (20), turnip (19), cranberry (14), and fructose (14). For certain cancers, we found the diets predicted to be anti-correlated include more cancer-related pathways than other diets. Taking esophageal squamous cell carcinoma (ESCC) as an example, 9 diets (e.g., tofu, persimmon peel, etc.) showed anti-correlated relationships, for which 11 of 52 significantly enriched pathways are ESCC-related (i.e., pathways also significantly enriched in ESCC). On the other hand, only 6 of 51 enriched pathways for other diets are ESCC-related. Pathways related to cell cycle, amino sugar and nucleotide sugar metabolism, biosynthesis of unsaturated fatty acids, protein export and spliceosome were observed to be enriched only for the anti-correlated diets. In conclusion, in addition to many associations not previously reported, our analysis recovered several experimentally validated relationships in which diet may exhibit a beneficial effect on the development of a disease, demonstrating the effectiveness of the ES method.

Cranberry showed the largest number of correlated relationships with diseases (40), with the highest enrichment scores against infection of the central nervous system (disease ID: 44, ES = 0.284), Crohn's disease (disease ID: 25, ES = 0.234) and glioblastoma (disease ID: 50, ES = 0.218) (Table S5). Kiwifruit followed cranberry in the list of foods with the most correlated relationships (39) against, e.g., ankylosing spondylitis (ES = 0.254), periodontitis (ES = 0.207) and bipolar disorder (ES = 0.203), among others. Caffeinated coffee was found to be correlated with lung cancer in our analysis, which is consistent with previous findings that coffee consumption may be associated with an increased risk of lung cancer (Guertin et al., 2015; Xie et al., 2016). A lard-rich diet was correlated with ovarian cancer (disease ID: 79, ES = 0.060), nonlymphocytic leukemia (disease ID: 3, ES = 0.065) and hepatocellular carcinoma (HCC) (disease ID: 54, ES = 0.047). Studies have reported that increased consumption of lard (an animal fat) may contribute to an elevated risk of ovarian cancer (Zhang et al., 2002; Fontelles et al., 2016). Possible mechanisms of action include the association of animal fat with prolactin secretion (Fontelles et al., 2016), which affects the pituitary secretion of trophic hormones (Melmed, 2011). Another example is the correlation of a fructose-rich diet with ovarian cancer (disease ID: 79, ES = 0.149), papillary thyroid carcinoma (disease ID: 51, ES = 0.098) and glioblastoma (disease ID: 50, ES = 0.083).

Alcohol had the most associations in our analysis (85) and, the most anti-correlated relationships with diseases (46). Alcohol was found to be anti-correlated with RA (ES = −0.114), whereas prostate cancer also displayed an anti-correlation with alcohol consumption (ES = −0.096).

**Figure 4:**
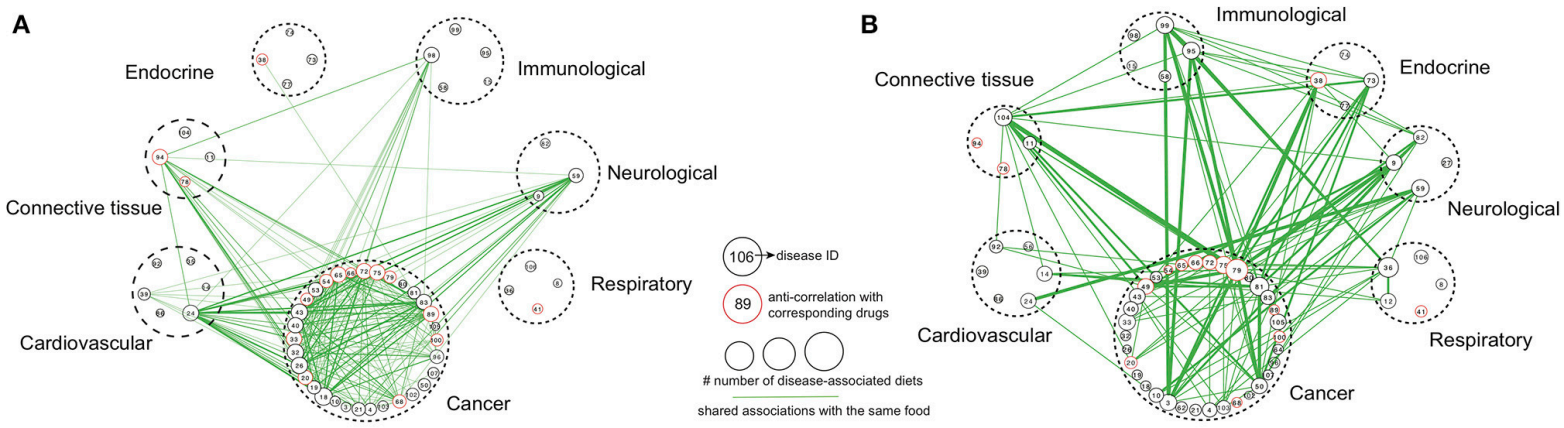
Disease class-centric association networks. Disease class-centric association networks built based on shared foods with same **(A)** anti-correlation or **(B)** correlation. The size of each node is proportional to the number of diets showing anti-correlated or correlated relationships with the corresponding disease. Diseases classified into same disease class are encircled by a dashed line. Edges connect diseases that were anti-correlated (or correlated) with the same foods, and the edge thickness is proportional to the number of such common foods. Only disease classes that have at least three diseases showing anti-correlated (or correlated) relationships with diet and edges with at least 4 associated foods are shown here.

To analyze diet-disease relationships from the disease angle, we subsequently linked disease pairs on the basis of shared foods with the same associations (either anti-correlation or correlation) between them, thereby generating two networks (Figures 4A,B, respectively). For clarity, the network construction did not include disease classes with fewer than 3 diseases showing anti-correlated (or correlated) relationships with diet or edges connecting diseases that had fewer than 4 shared foods. For several diseases that our analysis revealed to have either an anti-correlation or correlation with specific foods, the corresponding drug also showed an anti-correlation based on the enrichment score (Figures 4A,B). Examples included “altretamine-ovarian cancer” (cancer), “orciprenaline-emphysema” (respiratory), “gliquidone-diabetes” (endocrine) and “ketoprofen-rheumatoid arthritis” (connective tissue), among others, which also increased our confidence that the foods found to be anti-correlated with these diseases may exhibit positive effects. Blueberry, caffeinated coffee and turnip showed an anti-correlated relationship with ovarian cancer, with enrichment scores higher than altretamine (Table 1).

**Table 1:** Enrichment score comparisons between anti-correlated diet and recovered known drugs against diseases.

**Disease name**	**Drug**		**Diet**	
	**Drug name**	**Enrichment score**	**Food name**	**Enrichment score**
Ovarian cancer	Altretamine	−0.086	Blueberry	−0.139
			Caffeinated coffee	−0.125
			Turnip	−0.100
			Soy	−0.083
			Garlic oil	−0.067
Emphysema	Orciprenaline	−0.066	Alcohol	−0.102
			Persimmon peel	−0.073
			Orange	−0.068
Diabetes	Gliquidone	−0.138	Red wine	−0.174
			Blueberry	−0.142
			Caffeinated coffee	−0.125
			Turnip	−0.048
Rheumatoid arthritis	Ketoprofen	−0.067	Alcohol	−0.114
			Orange	−0.106
			Red wine	−0.079
			Cranberry	−0.079
			Garlic oil	−0.070
			Fructose	−0.043
			Tofu	−0.044
			Persimmon peel	−0.045

As shown in Figure 4A, many cancers were linked to each other. Non-small cell lung cancer (NSCLC, disease ID: 75) showed anti-correlations with 8 foods (including whey, soy and tofu). Moreover, NSCLC had high connectivity; it shared foods with anti-correlated profiles against 27 other cancers as well as (interestingly) with 46 non-cancer diseases (Figure 4A). PDAC (disease ID: 83) was anti-correlated with 8 foods (including tofu and persimmon peel) and was linked to 31 other cancers as well as 54 non-cancer diseases (Figure 4A). However, there were also several cases with highly unique disease gene expression signatures, producing an isolated position in the network. Among such cases were respiratory-related diseases; the foods associated with these conditions were rarely anti-correlated with any other disease (Figure 4A).

In the network of correlated relationships between diet and diseases (Figure 4B), several diseases had anti-correlated relationships with their corresponding drugs, highlighting that foods correlated with a specific disease could pose a risk. Examples included “turnip-osteoarthritis” and “grape-uterine fibroids.” An examination of the network connectivity revealed a similar pattern as above; many cancers were intra- and interconnected with non-cancer diseases (Figure 4B). One disease that presented high connectivity in the network was ovarian cancer. Ovarian cancer (disease ID: 79) showed correlated relationships with 10 diets (including lard-rich, fructose-rich and high-fat diets) and was connected with 25 other cancers as well as (interestingly) with 53 non-cancer diseases (Figure 4B). We think that the relationships revealed between specific diseases or disease classes, which were often unexpected, highlight the need for a complete assessment of an individual's medical status before proceeding to dietary recommendations.

### A comparative view of diet-disease and drug-disease associations based on global gene expression signatures

**Figure 5:**
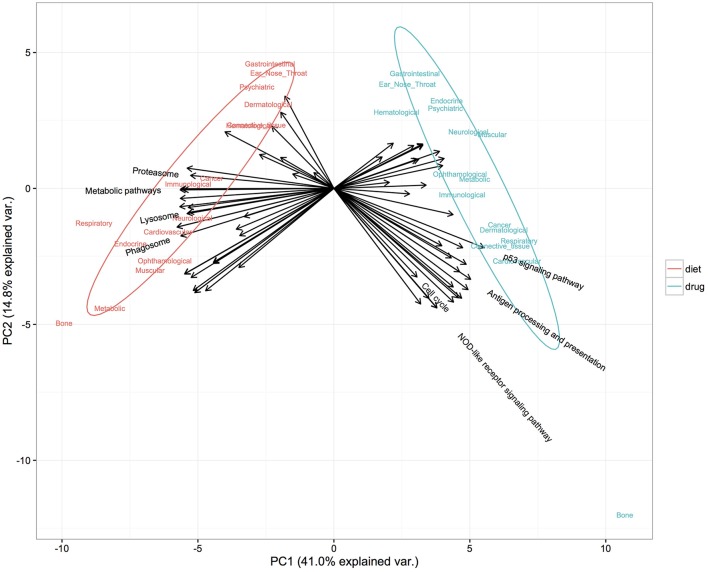
Distinct pathways enriched in diet and drug gene expression signatures found anti-correlated with diseases. The first two components from PCA accounted for 55.8% of the total variance. Colors denote two classes of administrations: diet (red) and drug (blue). Arrows denote representative variables (pathways) with a relative contribution ranked in the top 30 to either of the principal components and are labeled with pathway names.

Both diet and drugs play important roles in disease prevention; however, their different modes of action against diseases have not been fully evaluated. Here, we investigated and compared the different underlying mechanisms of diet and drugs in terms of affected biological pathways and chemical structural similarity between their components. First, a principal component analysis based on significantly enriched pathways (FDR < 0.05) (see Materials and Methods for more details) was applied to evaluate differences in the mechanisms by which diets and drugs induced their positive effect on diseases. Only diets and drugs showing an anti-correlated profile with a disease based on the ES were included. Figure 5 clearly demonstrates that diet and drugs had distinctly separate significantly enriched pathways. Pathways related to metabolism, including metabolic pathways, phagosome and lysosome, tended to be enriched for diet. In contrast, pathways related to cell signaling, including the P53 signaling pathway, cell cycle and NOD-like receptor signaling pathway, were mainly enriched for drugs.

In a further examination of different preventive actions against diseases between diet and drugs at the pathway level, our attention was restricted to enriched pathways for diet (or drugs) that were also altered in diseases (simply referred to as disease-related pathways). Using the same method as above, we identified and extracted, for each disease class, the top three disease-related pathways enriched in the most anti-correlated foods (and drugs). As described above, pathways enriched for diet were mainly related to metabolism, such as oxidative phosphorylation, arginine and proline metabolism, while pathways enriched for drugs tended to be associated with signaling processes (Table S6). This discrimination between the main target pathways elicited by these two classes of administration is in agreement with published findings that diet mainly affects metabolic pathways (Mattson and Shea, 2003; Pi-Sunyer, 2005; Pavlova and Thompson, 2016) while drugs affect signaling pathways (Ono and Han, 2000; Downward, 2003; Fresno Vara et al., 2004; Nencioni et al., 2007; Klaus and Birchmeier, 2008).

In addition to biological functions, we compared foods and drugs from a chemical structural point of view. Only plant-based foods with phytochemical compositions available in NutriChem (Jensen et al., 2015b) were included in this comparison. In total, we retrieved 20 food-drug pairs in which at least one compound in the food showed chemical structural similarity to a drug (see Materials and Methods section). Interestingly, compared with other food-drug pairs, these pairs tended to share more anti-correlated diseases identified by the ES method (Figure S2); however, this result was not statistically significant. For example, coconut and azacitidine shared 10 anti-correlated diseases, such as bladder cancer, Huntington's disease and cardiomyopathy. Coconut contains kinetin riboside, a molecule with structural similarity to azacitidine. Similarly, garlic had 8 overlapping anti-correlated diseases with the drugs chlorpromazine and mesoridazine, which are structurally similar to trifluoperazine (derived from garlic). Although the size of the dataset was relatively small for forming general conclusions, we should not reject the idea that, despite the complex chemical composition of foods, one critical bioactive component may sometimes “drive” the gene expression response of the host.

### Evaluating the therapeutic value of diet based on an integrated topology analysis

**Figure 6:**
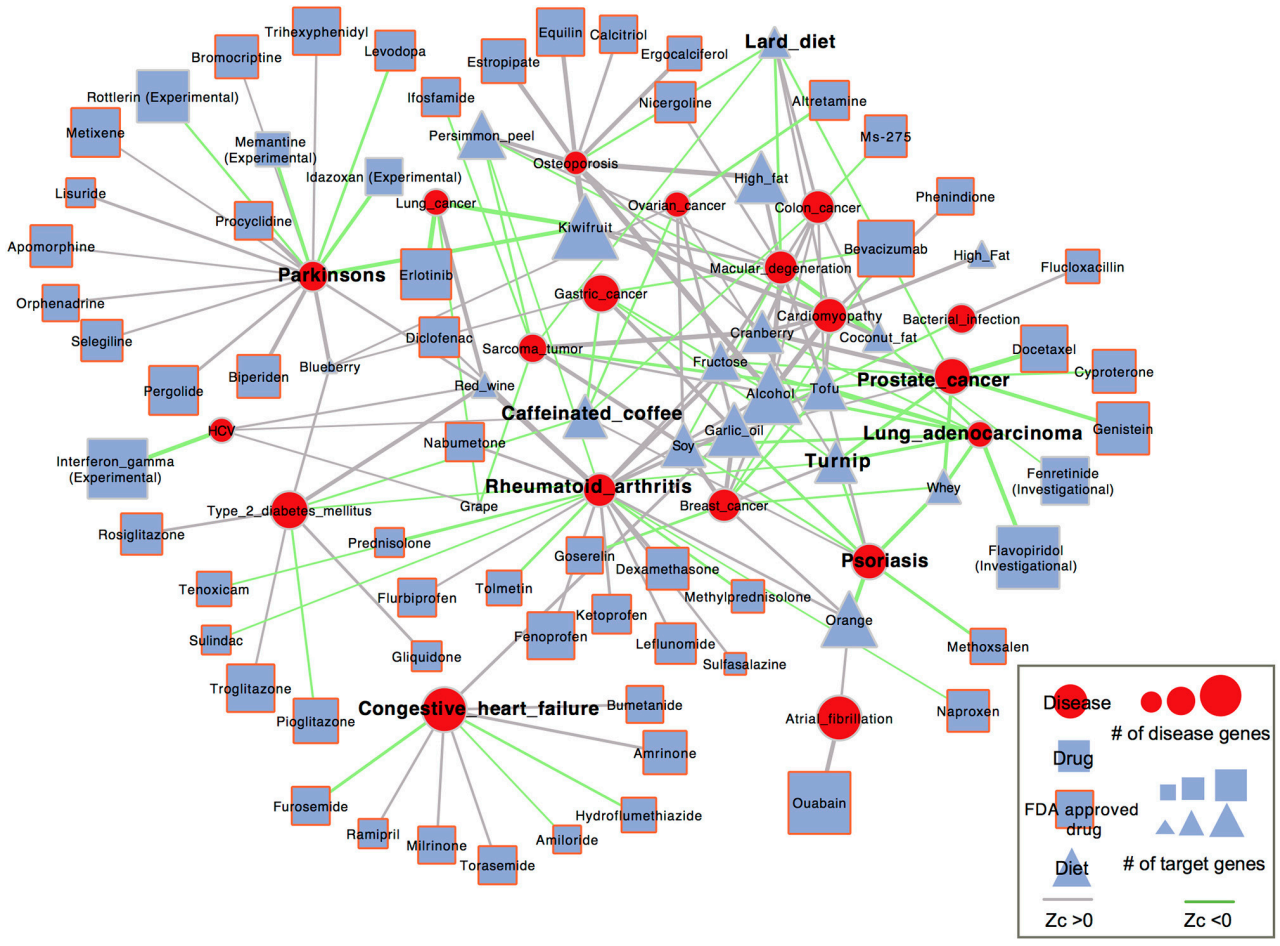
Network-based diet-disease and drug-disease proximity. Nodes represent diseases (red circle), diet (blue square) or drug (blue triangle). Blue triangle with red border represents FDA approved drugs. The node size scales with the number of disease genes and the number of DE genes of diet and drug. Diets (or drugs) and diseases are linked by anti-correlated relationships between them. The link color represents whether the diet (or drug) is proximal (Z_c_ < 0, green) or distant (Z_c_ < 0, gray) to the disease. The edge thickness is proportional to the absolute value of the proximity score.

Our analysis here was based on the hypothesis that if a drug is effective against a disease, it may target proteins within or in close proximity to the disease gene module. Accordingly, we calculated proximity scores, Z_*c*_, from the module of differentially expressed (DE) genes of diet to the module of disease-related genes in the PPI network. To put our findings into perspective, we compared diets with drugs in terms of network proximity. Therefore, proximity scores between FDA-approved (and clinically investigated) drugs and corresponding diseases were also calculated. Based on diet-disease anti-correlation relationships that we identified above and information on disease modules retrieved from Guney et al. (2016), 19 diseases, 18 foods and 63 drugs were involved in the network analysis and visualization (Figure 6).

We found that 40 out of the 98 (41%) diet-disease pairs identified as anti-correlated were closer than the distance from randomly selected sets of proteins to the disease genes (Z_c_ < 0), only slightly lower than 28 of the 62 (45%) known drug-disease associations (Table S7). These 40 diet–disease pairs, whose associations were supported by both the ES and proximity method, probably represent a more trustworthy set for dietary interventions. In addition, we investigated whether there were significant differences between the proximity scores of diet-disease and drug-disease pairs. The diets identified as anti-correlated with diseases showed similar topological proximity to the disease proteins as the corresponding drugs (Fisher's exact test, *P* = 0.6247). More specifically, for some diseases, such as colon cancer, lung cancer, lung adenocarcinoma, ovarian cancer and HCV, the gene expression changes induced by the corresponding drugs were much closer to the disease genes than all the anti-correlated foods. On the other hand, several foods that were anti-correlated with certain diseases induced gene expression changes that were much closer to the disease genes than the corresponding drugs; garlic oil was closer to bacterial infection (Z_c_ = −0.57) than flucloxacillin (Z_c_ = 0.94), and kiwifruit (Z_c_ = −2.12) was much closer to Parkinson's disease than all FDA-approved drugs. Garlic oil has been reported to have an antimicrobial effect against human enteric bacteria (Aydin et al., 2000; Ross et al., 2001). Melatonin, a derivative of the essential amino acid tryptophan identified in kiwifruit, was demonstrated to mitigate Parkinson's disease (Wang, 2009). As shown in Figure 6, turnip and whey were connected to most diseases with Z_c_ < 0, such as psoriasis and prostate cancer. The proximity between all corresponding FDA-approved drugs and these two diseases showed Z_c_ < 0, indicating a possible explanation of their efficacy. Notably, for the same diseases, both turnip and whey had comparable proximity scores to the corresponding drugs. The proximal distance in the whey-psoriasis pair was Z_c_ = −1.85; it has been reported that XP-828L, a protein extract isolated from bovine sweet whey, has potential benefit for mild to moderate psoriasis (Drouin et al., 2007). Whey was also proximal to prostate cancer (Z_c_ = −1.66) and has been shown to increase the synthesis of glutathione (GSH), which may play an important role in preventing prostate cancer development through its antioxidant activities (Kent et al., 2003). Another food, orange, was proximal to psoriasis (Z_c_ = −2.26), and its peel extracts have been demonstrated in a pilot study to significantly reduce the skin lesions of patients (Hakim et al., 2000).

### *In silico* identification of synergistic food pairs

Because humans normally do not consume foods in isolation and due to possible interactions between different foods, we employed gene expression profiles to identify food pairs with synergistic effects (signal argumentation when consumed together). Bansal et al. (2014) evaluated and ranked 31 computational methods used to predict the activities of compound pairs based on an experimentally assessed gold standard. We selected as candidates three methods that ranked at the top of Bansal's list for identifying synergistic pairs (see Materials and Methods for more details). Analogously, the literature-reported food-disease relationships were used for assessing the suitability of these approaches in our study. Here, we used 4 food pairs (consisting of 5 different foods) as a gold standard (see Materials and Methods). When we applied these three methods to the gene expression profiles of the 5 foods, we recovered 3, 0, and 1 gold standard food pairs using Rank 2, 4, and 9 methods, respectively. Notably, the Rank 2 method recovered 3 out of 4 gold standard food pairs, which was comparable to the PC index (which essentially quantifies the proportion of corrected identification) of 0.605 reported in Bansal et al. (2014). Therefore, the Rank 2 method was used to identify potential pairwise synergistic relationships between the 18 foods in our study. As a result, 58 food pairs with synergistic effects were identified (Figure S4; Table S8).

We found that food pairs predicted to be synergistic tend to share more anti-correlated diseases (Wilcox ranked sum test, *P* = 0.00018, Figure S5). As the pair with the highest interaction score, alcohol and kiwifruit had 23 anti-correlated diseases in common based on the ES. Persimmon peel and kiwifruit, with a relatively high interaction score, were anti-correlated with 31 and 44 diseases, respectively, while 23 diseases were shared by these two foods. Another pair with a high interaction score was orange-cranberry, which were both anti-correlated with cancers, including chronic myeloid leukemia, NSCLC and autoimmune disease rheumatoid arthritis. We also found that they shared 4 significantly enriched pathways mainly related to immune response, including ribosome, phagosome, shigellosis and pathogenic *Escherichia coli* infection.

In addition, for synergistic food pairs, we investigated the correlations of their ES profiles across all 111 diseases included in our dataset. As a result, 42 out of 58 synergistic pairs showed positive correlations, among which 31 pairs were significantly positively correlated (Pearson correlation, *P* < 0.05). In contrast, only 4 of 16 pairs that showed negative correlations were statistically significant. This finding also reflects the similar therapeutic potential against diseases between the synergistic food pairs.

## Discussion

In summary, we developed a computational framework using genome-wide gene-expression profiles and network-based features to identify the effects of diet on disease development and/or progression. Previous studies on drug repositioning proposed different methods to establish drug-disease correlations using gene expression signatures and evaluated their performance by their ability to recover known relationships (Sirota et al., 2011; Shigemizu et al., 2012). Here, the enrichment score method developed by Pacini et al. (2013) was evaluated in a larger dataset before being applied to food-induced gene expression signatures. The recovery percentage was similar to a previous study on drug-repositioning (Shigemizu et al., 2012), and the cancer disease class had a significantly higher recovery rate using the ES method than random.

We were also able to recover a significant number of cases in which a particular food has been experimentally shown to have a positive or negative effect on disease progression. Hierarchical clustering based on correlation profiles of each disease across the whole drug or food panel led to the identification of several disease clusters with potential common pathophysiological properties, with the cancer cluster to be the most striking example. Our result is consistent with reports that cancers in different organs may have underlying biological similarities (Yang and Sun, 2007; Risbridger et al., 2010; Dawany et al., 2011). This finding increased our confidence that also the new diet-disease associations that our *in silico* analysis proposed should be carefully examined and perhaps followed up for experimental validation. From our analysis, kiwi, tofu, soy and persimmon all stand out as the diets with most anti-correlation relationships with cancers. Due to its antioxidant and cytotoxic properties, kiwifruit has been used to treat cancers in Chinese traditional medicine (Motohashi et al., 2002). Additionally, kiwifruit has a protective effect against cardiovascular disease as a result of its antioxidative, antihypertensive and hypocholesterolemic activities (Jung et al., 2005). Tofu is a primary soy food that is commonly consumed in Asian countries, such as China, Japan and Korea, which have been reported to have low incidences of breast and prostate cancer (Adlercreutz, 2002; Kim et al., 2008). Soy products contain isoflavones; these phytoestrogens, such as genistein, daidzein and glycitein, are known to influence several biological processes, including the biological activity of sex hormones and their effect on growth factors (Adlercreutz, 2002). There is increasing evidence that soy has a protective effect on non-small cell lung cancer (NSCLC) cases with epidermal growth factor receptor (EGFR) mutations and breast, ovarian and prostate cancers (Shu et al., 2001; Lee et al., 2003; Zhang et al., 2004; Matsuo et al., 2008; Mishra et al., 2011). In particular, tofu has been reported to be associated with a decreased risk of breast cancer (Hirose et al., 2003; Kim et al., 2008). In addition, tofu intake could alter susceptibility to low-density lipoprotein oxidation and may lower the risk of coronary heart disease (Ashton et al., 2000). Persimmon peel is rich in antioxidants, such as carotenoids and polyphenols (Gorinstein et al., 2001; Izuchi et al., 2009), which are known to protect against oxidative stress and have been implicated in various pathological conditions, including cardiovascular disease, cancer and neurological disorders (Dalle-Donne et al., 2006; Valko et al., 2007).

Similar to identifying the beneficial effects of specific foods, the ES method seemed to successfully recover known negative effects of diet in the development or progression of diseases, and it generated new relationships that should be considered for experimental validation or further computational analysis. One notable example is the correlation of fructose diet with several forms of cancer. The intake of dietary sugars such as glucose and fructose has increased considerably, especially fructose, which is frequently used as food additive for palatability. Furthermore, it has been reported that fructose promotes cancer growth by a variety of mechanisms, including altered cell metabolism and indirect enhancement of protein synthesis (Port et al., 2012), increased reactive oxygen species, DNA damage and inflammation (Liu and Heaney, 2011). Alcohol is probably the most intriguing case, since in our analysis it had the most associations (including correlations and anti-correlations) and, surprisingly, the most anti-correlated relationships with diseases. Usually, alcohol intake is considered harmful to human health; however, alcohol consumption has also been associated with a lower risk of specific diseases (Howard et al., 2004; Allen et al., 2009; Kallberg et al., 2009; Costanzo et al., 2010; Brien et al., 2011). Here, alcohol was predicted to be anti-correlated with RA, which is consistent with the finding of different studies that increased alcohol intake decreased the risk of RA (Kallberg et al., 2009; Maxwell et al., 2010; Di Giuseppe et al., 2012; Jin et al., 2014). Prostate cancer also displayed an anti-correlation with alcohol consumption, whereas similar anti-correlated relationships between prostate cancer and alcohol, or specific alcohol types such as red wine, have been reported previously (Breslow et al., 1999; Schoonen et al., 2005).

Diet may not only exert beneficial effects through the directly affected proteins but may also propagate their effects on human health though functionally or physically related genes, especially disease-related ones, within a human protein-protein interaction (PPI) network. To further quantify the therapeutic effects of diets that were identified as anti-correlated with diseases using the global-level ES method, we used the relative proximity measure proposed by Guney et al. (2016) to capture topological features in the PPI network. In several cases, the therapeutic potential of particular diets was further supported by a network-based topological analysis, which revealed that foods often directly target genes within or in the vicinity of specific disease gene modules in the interactome and are likely to have comparable therapeutic effects with the corresponding drugs. Compared to the ES method that reflects the global effects of a dietary intervention, the network-based proximity method, which depends on the existing knowledge about disease proteins and a set of gene expression signatures, could provide additional insight into the relationships between diet and diseases. However, as Guney et al. postulated (Guney et al., 2016), there are some limitations of network-based proximity. The method was unable to explain the therapeutic value of cases where the drugs (or in our case foods) were distant to the corresponding diseases, nor could it predict the direction of the effect (beneficial or harmful) of drugs (or foods). The gene expression anti-correlation and network topology-based approaches focus on different angles in quantifying the possible therapeutic effects, and they are less likely to correlate with each other (Figure S3); therefore, the proximity score method can still be considered as a complementary tool for uncovering the underlying mechanism of the therapeutic value of dietary interventions. From our integrative analysis, the list of “recommended diet for corresponding diseases” with top evidence (both high enrichment scores and close proximity to disease genes) include orange, whey and coconut fat for psoriasis, lung adenocarcinoma and macular degeneration, respectively. In addition to the aforementioned correlated foods with literature support, other identified foods with relatively stronger evidence include fructose-rich diet for chronic intermittent hypoxia and ovarian cancer, as well as alcohol for osteonecrosis of the jaw and sarcoidosis, which should be avoided during the course of corresponding diseases.

## Conclusions

Despite our identified anti-correlated and avoided foods, the prospective personalized nutritional recommendation needs deeper understanding and well-designed systematic analysis of more experimental data, and metadata information. In our study, large-scale public gene expression data for drugs and diseases were integrated into a comprehensive analysis. These data resources have been extensively used for drug repositioning to screen for promising drugs with new therapeutic indications, which highlights the efficacy of a systematic analysis of a large number of gene expression profiles. In contrast to drugs and other bioactive molecules for which a large number of gene expression profiles from cell lines, animal models and humans exist, the publicly available gene expression data for diet are very limited (only 18 foods were available to use by February 2016). It will certainly increase the reliability and usage of our proof-of-concept study if gene expression profiles from more foods are incorporated in the analysis, and this is a direction that the scientific community should take into consideration. Furthermore, with more public data and relevant sample information, multivariable analyses can be conducted to obtain a more complete description of the effects of diet in different groups of people. It should be also noted that the enrichment score method to establish diet-disease correlations is based on global gene expression profiles, thus certain diseases that induce sporadic or local expression variations might not be suited to such analysis. Furthermore, our identified food pairs were predictions based on well-established methods in another field, thus they should be subject to appropriate experimental and clinical validations before further clinical applications.

## Author contributions

TZ, YN, GP involved in the conception and designed of the study. TZ, YN processed samples and analyzed the data. TZ, YN, GP wrote the manuscript. All authors reviewed and revised the manuscript. All authors read and approved the final manuscript.

### Conflict of interest statement

The authors declare that the research was conducted in the absence of any commercial or financial relationships that could be construed as a potential conflict of interest.

## Supplementary material

### Availability of data and materials

The raw and processed gene expression profiles of foods and diseases are available in NCBI GEO and ArrayExpress (see Tables S1, S2); The gene expression profiles for drugs are available in NCBI GEO, ArrayExpress, Cmap and DrugMatrix® (https://ntp.niehs.nih.gov/drugmatrix/index.html) (see Table S3).

## Abbreviations

AD: Alzheimer's disease
ALL: acute lymphoblastic leukemia
AML: acute myeloid leukemia
AMPK: adenosine monophosphate (AMP)-activated protein kinase
BE: Barrett's esophagus
CD: Crohn's disease
CML: chronic myelogenous leukemia
COPD: chronic obstructive pulmonary disease
DAPH, 4: 5-dianilinophthalimide
DE: differentially expressed
EAC: Esophageal adenocarcinoma
EGFR: epidermal growth factor receptor
ES: enrichment score
ESCC: esophageal squamous cell carcinoma
HBV: hepatitis B virus
HBV-ALF: acute liver failure due to hepatitis B virus
HBV-associated HCC: hepatitis B virus-associated hepatocellular carcinoma
HCC: hepatocellular carcinoma
HCV: hepatitis C virus
IBD: inflammatory bowel disease
JIA: juvenile rheumatoid arthritis
mTOR: mechanistic target of rapamycin
NSCLC: non-small cell lung cancer
PCA: principal component analysis
PDAC: pancreatic ductal adenocarcinoma
PPI: protein-protein interaction
RA: rheumatoid arthritis
SLE: systemic lupus erythematosus
UC: ulcerative colitis.

## References

[B1] Adlercreutz H. (2002). Phyto-oestrogens and cancer. Lancet Oncol. 3, 364–373. 10.1016/S1470-2045(02)00777-5 12107024

[B2] Albenberg L. G. Wu G. D. (2014). Diet and the intestinal microbiome: associations, functions, and implications for health and disease. Gastroenterology 146, 1564–1572. 10.1053/j.gastro.2014.01.058 24503132 PMC4216184

[B3] Allen N. E. Beral V. Casabonne D. Kan S. W. Reeves G. K. Brown A.. (2009). Moderate alcohol intake and cancer incidence in women. J. Natl. Cancer Inst. 101, 296–305. 10.1093/jnci/djn514 19244173

[B4] Andersson A. Olofsson T. Lindgren D. Nilsson B. Ritz C. Eden P.. (2005). Molecular signatures in childhood acute leukemia and their correlations to expression patterns in normal hematopoietic subpopulations. Proc. Natl. Acad. Sci. U.S.A. 102, 19069–19074. 10.1073/pnas.0506637102 16354839 PMC1323166

[B5] Ashton E. L. Dalais F. S. Ball M. J. (2000). Effect of meat replacement by tofu on CHD risk factors including copper induced LDL oxidation. J. Am. Coll. Nutr. 19, 761–767. 10.1080/07315724.2000.10718067 11194529

[B6] Aydin A. Ersoz G. Tekesin O. Akcicek E. Tuncyurek M. (2000). Garlic oil and Helicobacter pylori infection. Am. J. Gastroenterol. 95, 563–564. 10.1016/S0002-9270(99)00871-0 10685782

[B7] Badimon L. Vilahur G. Padro T. (2016). Systems biology approaches to understand the effects of nutrition and promote health. Br. J. Clin. Pharmacol. 83, 38–45. 10.1111/bcp.12965 27062443 PMC5338131

[B8] Bansal M. Yang J. Karan C. Menden M. P. Costello J. C. Tang H.. (2014). A community computational challenge to predict the activity of pairs of compounds. Nat. Biotechnol. 32, 1213–1222. 10.1038/nbt.3052 25419740 PMC4399794

[B9] Barres R. Zierath J. R. (2016). The role of diet and exercise in the transgenerational epigenetic landscape of T2DM. Nat. Rev. Endocrinol. 12, 441–451. 10.1038/nrendo.2016.87 27312865

[B10] Bolton E. E. Wang Y. Thiessen P. A. Bryant S. H. (2008). PubChem: integrated platform of small molecules and biological activities. Annu. Rep. Comput. Chem. 4, 217–241. 10.1016/S1574-1400(08)00012-1

[B11] Breitling R. Armengaud P. Amtmann A. Herzyk P. (2004). Rank products: a simple, yet powerful, new method to detect differentially regulated genes in replicated microarray experiments. FEBS Lett. 573, 83–92. 10.1016/j.febslet.2004.07.055 15327980

[B12] Breslow R. A. Wideroff L. Graubard B. I. Erwin D. Reichman M. E. Ziegler R. G.. (1999). Alcohol and prostate cancer in the NHANES I epidemiologic follow-up study. first national health and nutrition examination survey of the United States. Ann. Epidemiol. 9, 254–261. 10.1016/S1047-2797(98)00071-4 10332931

[B13] Brien S. E. Ronksley P. E. Turner B. J. Mukamal K. J. Ghali W. A. (2011). Effect of alcohol consumption on biological markers associated with risk of coronary heart disease: systematic review and meta-analysis of interventional studies. BMJ 342:d636. 10.1136/bmj.d636 21343206 PMC3043110

[B14] Costanzo S. Di Castelnuovo A. Donati M. B. Iacoviello L. de Gaetano G. (2010). Alcohol consumption and mortality in patients with cardiovascular disease: a meta-analysis. J. Am. Coll. Cardiol. 55, 1339–1347. 10.1016/j.jacc.2010.01.006 20338495

[B15] Dalle-Donne I. Rossi R. Colombo R. Giustarini D. Milzani A. (2006). Biomarkers of oxidative damage in human disease. Clin. Chem. 52, 601–623. 10.1373/clinchem.2005.061408 16484333

[B16] David L. A. Maurice C. F. Carmody R. N. Gootenberg D. B. Button J. E. Wolfe B. E.. (2014). Diet rapidly and reproducibly alters the human gut microbiome. Nature 505, 559–563. 10.1038/nature12820 24336217 PMC3957428

[B17] Dawany N. B. Dampier W. N. Tozeren A. (2011). Large-scale integration of microarray data reveals genes and pathways common to multiple cancer types. Int. J. Cancer 128, 2881–2891. 10.1002/ijc.25854 21165954

[B18] Di Giuseppe D. Alfredsson L. Bottai M. Askling J. Wolk A. (2012). Long term alcohol intake and risk of rheumatoid arthritis in women: a population based cohort study. BMJ 345:e4230. 10.1136/bmj.e4230 22782847 PMC3393782

[B19] Dowell R. D. (2011). The similarity of gene expression between human and mouse tissues. Genome Biol. 12:101. 10.1186/gb-2011-12-1-101 21241524 PMC3091293

[B20] Downward J. (2003). Targeting RAS signalling pathways in cancer therapy. Nat. Rev. Cancer 3, 11–22. 10.1038/nrc969 12509763

[B21] Drouin R. Lamiot E. Cantin K. Gauthier S. F. Pouliot Y. Poubelle P. E.. (2007). XP-828L (Dermylex), a new whey protein extract with potential benefit for mild to moderate psoriasis. Can. J. Physiol. Pharmacol. 85, 943–951. 10.1139/Y07-084 18066141

[B22] Edgar R. Domrachev M. Lash A. E. (2002). Gene Expression Omnibus: NCBI gene expression and hybridization array data repository. Nucleic Acids Res. 30, 207–210. 10.1093/nar/30.1.207 11752295 PMC99122

[B23] Falcon S. Gentleman R. (2007). Using GOstats to test gene lists for GO term association. Bioinformatics 23, 257–258. 10.1093/bioinformatics/btl567 17098774

[B24] Finkel T. (2015). The metabolic regulation of aging. Nat. Med. 21, 1416–1423. 10.1038/nm.3998 26646498

[B25] Fontelles C. C. Guido L. N. Rosim M. P. Andrade Fde O. Jin L. Inchauspe J.. (2016). Paternal programming of breast cancer risk in daughters in a rat model: opposing effects of animal- and plant-based high-fat diets. Breast Cancer Res. 18:71. 10.1186/s13058-016-0729-x 27456846 PMC4960664

[B26] Fortney K. Griesman J. Kotlyar M. Pastrello C. Angeli M. Sound-Tsao M.. (2015). Prioritizing therapeutics for lung cancer: an integrative meta-analysis of cancer gene signatures and chemogenomic data. PLoS Comput. Biol. 11:e1004068. 10.1371/journal.pcbi.1004068 25786242 PMC4364883

[B27] Fresno Vara J. A. Casado E. de Castro J. Cejas P. Belda-Iniesta C. Gonzalez-Baron M. (2004). PI3K/Akt signalling pathway and cancer. Cancer Treat. Rev. 30, 193–204. 10.1016/j.ctrv.2003.07.007 15023437

[B28] Ganter B. Tugendreich S. Pearson C. I. Ayanoglu E. Baumhueter S. Bostian K. A.. (2005). Development of a large-scale chemogenomics database to improve drug candidate selection and to understand mechanisms of chemical toxicity and action. J. Biotechnol. 119, 219–244. 10.1016/j.jbiotec.2005.03.022 16005536

[B29] Gilson M. K. Liu T. Baitaluk M. Nicola G. Hwang L. Chong J. (2016). BindingDB in 2015: a public database for medicinal chemistry, computational chemistry and systems pharmacology. Nucleic Acids Res. 44, D1045–D1053. 10.1093/nar/gkv1072 26481362 PMC4702793

[B30] Goh K. I. Cusick M. E. Valle D. Childs B. Vidal M. Barabasi A. L. (2007). The human disease network. Proc. Natl. Acad. Sci. U.S.A. 104, 8685–8690. 10.1073/pnas.0701361104 17502601 PMC1885563

[B31] Gorinstein S. Zachwieja Z. Folta M. Barton H. Piotrowicz J. Zemser M.. (2001). Comparative contents of dietary fiber, total phenolics, and minerals in persimmons and apples. J. Agric. Food Chem. 49, 952–957. 10.1021/jf000947k 11262055

[B32] Guertin K. A. Freedman N. D. Loftfield E. Graubard B. I. Caporaso N. E. Sinha R. (2015). Coffee consumption and incidence of lung cancer in the NIH-AARP Diet and Health Study. Int. J. Epidemiol. 45, 929–939. 10.1093/ije/dyv104 26082405 PMC5005936

[B33] Guney E. Menche J. Vidal M. Barabasi A. L. (2016). Network-based *in silico* drug efficacy screening. Nat. Commun. 7:10331. 10.1038/ncomms10331 26831545 PMC4740350

[B34] Hakim I. A. Harris R. B. Ritenbaugh C. (2000). Citrus peel use is associated with reduced risk of squamous cell carcinoma of the skin. Nutr. Cancer 37, 161–168. 10.1207/S15327914NC372_7 11142088

[B35] Hirose K. Takezaki T. Hamajima N. Miura S. Tajima K. (2003). Dietary factors protective against breast cancer in Japanese premenopausal and postmenopausal women. Int. J. Cancer 107, 276–282. 10.1002/ijc.11373 12949807

[B36] Hong F. Breitling R. McEntee C. W. Wittner B. S. Nemhauser J. L. Chory J. (2006). RankProd: a bioconductor package for detecting differentially expressed genes in meta-analysis. Bioinformatics 22, 2825–2827. 10.1093/bioinformatics/btl476 16982708

[B37] Howard A. A. Arnsten J. H. Gourevitch M. N. (2004). Effect of alcohol consumption on diabetes mellitus: a systematic review. Ann. Intern. Med. 140, 211–219. 10.7326/0003-4819-140-6-200403160-00011 14757619

[B38] Iorio F. Bosotti R. Scacheri E. Belcastro V. Mithbaokar P. Ferriero R.. (2010). Discovery of drug mode of action and drug repositioning from transcriptional responses. Proc. Natl. Acad. Sci. U.S.A. 107, 14621–14626. 10.1073/pnas.1000138107 20679242 PMC2930479

[B39] Iorio F. Rittman T. Ge H. Menden M. Saez-Rodriguez J. (2013). Transcriptional data: a new gateway to drug repositioning? Drug Discov. Today 18, 350–357. 10.1016/j.drudis.2012.07.014 22897878 PMC3625109

[B40] Irizarry R. A. Bolstad B. M. Collin F. Cope L. M. Hobbs B. Speed T. P. (2003). Summaries of Affymetrix GeneChip probe level data. Nucleic Acids Res. 31:e15. 10.1093/nar/gng015 12582260 PMC150247

[B41] Iskar M. Zeller G. Blattmann P. Campillos M. Kuhn M. Kaminska K. H.. (2013). Characterization of drug-induced transcriptional modules: towards drug repositioning and functional understanding. Mol. Syst. Biol. 9:662. 10.1038/msb.2013.20 23632384 PMC3658274

[B42] Izuchi R. Takahashi H. Inada Y. (2009). Preparing a carotenoid polyphenol-enriched extract from the peel of persimmon, Diospyros kaki L.f. Biosci. Biotechnol. Biochem. 73, 2793–2795. 10.1271/bbb.90616 19966462

[B43] Jensen K. Ni Y. Panagiotou G. Kouskoumvekaki I. (2015a). Developing a molecular roadmap of drug-food interactions. PLoS Comput. Biol. 11:e1004048. 10.1371/journal.pcbi.1004048 25668218 PMC4323218

[B44] Jensen K. Panagiotou G. Kouskoumvekaki I. (2014). Integrated text mining and chemoinformatics analysis associates diet to health benefit at molecular level. PLoS Comput. Biol. 10:e1003432. 10.1371/journal.pcbi.1003432 24453957 PMC3894162

[B45] Jensen K. Panagiotou G. Kouskoumvekaki I. (2015b). NutriChem: a systems chemical biology resource to explore the medicinal value of plant-based foods. Nucleic Acids Res. 43, D940–D945. 10.1093/nar/gku724 25106869 PMC4383999

[B46] Jin Z. Xiang C. Cai Q. Wei X. He J. (2014). Alcohol consumption as a preventive factor for developing rheumatoid arthritis: a dose-response meta-analysis of prospective studies. Ann. Rheum. Dis. 73, 1962–1967. 10.1136/annrheumdis-2013-203323 23897767

[B47] Johnson S. C. Rabinovitch P. S. Kaeberlein M. (2013). mTOR is a key modulator of ageing and age-related disease. Nature 493, 338–345. 10.1038/nature11861 23325216 PMC3687363

[B48] Jung K. A. Song T. C. Han D. Kim I. H. Kim Y. E. Lee C. H. (2005). Cardiovascular protective properties of kiwifruit extracts *in vitro*. Biol. Pharm. Bull. 28, 1782–1785. 10.1248/bpb.28.1782 16141561

[B49] Kallberg H. Jacobsen S. Bengtsson C. Pedersen M. Padyukov L. Garred P.. (2009). Alcohol consumption is associated with decreased risk of rheumatoid arthritis: results from two Scandinavian case-control studies. Ann. Rheum. Dis. 68, 222–227. 10.1136/ard.2007.086314 18535114 PMC2937278

[B50] Kent K. D. Harper W. J. Bomser J. A. (2003). Effect of whey protein isolate on intracellular glutathione and oxidant-induced cell death in human prostate epithelial cells. Toxicol. In Vitro 17, 27–33. 10.1016/S0887-2333(02)00119-4 12537959

[B51] Kim M. K. Kim J. H. Nam S. J. Ryu S. Kong G. (2008). Dietary intake of soy protein and tofu in association with breast cancer risk based on a case-control study. Nutr. Cancer 60, 568–576. 10.1080/01635580801966203 18791919

[B52] Klaus A. Birchmeier W. (2008). Wnt signalling and its impact on development and cancer. Nat. Rev. Cancer 8, 387–398. 10.1038/nrc2389 18432252

[B53] Knight E. M. Martins I. V. Gumusgoz S. Allan S. M. Lawrence C. B. (2014). High-fat diet-induced memory impairment in triple-transgenic Alzheimer's disease (3xTgAD) mice is independent of changes in amyloid and tau pathology. Neurobiol. Aging 35, 1821–1832. 10.1016/j.neurobiolaging.2014.02.010 24630364 PMC4024197

[B54] Lamb J. Crawford E. D. Peck D. Modell J. W. Blat I. C. Wrobel M. J.. (2006). The Connectivity Map: using gene-expression signatures to connect small molecules, genes, and disease. Science 313, 1929–1935. 10.1126/science.1132939 17008526

[B55] Law V. Knox C. Djoumbou Y. Jewison T. Guo A. C. Liu Y.. (2014). DrugBank 4.0: shedding new light on drug metabolism. Nucleic Acids Res. 42, D1091–D1097. 10.1093/nar/gkt1068 24203711 PMC3965102

[B56] Lee M. M. Gomez S. L. Chang J. S. Wey M. Wang R. T. Hsing A. W. (2003). Soy and isoflavone consumption in relation to prostate cancer risk in China. Cancer Epidemiol. Biomarkers Prev. 12, 665–668. 12869409

[B57] Lin S. J. Kaeberlein M. Andalis A. A. Sturtz L. A. Defossez P. A. Culotta V. C.. (2002). Calorie restriction extends Saccharomyces cerevisiae lifespan by increasing respiration. Nature 418, 344–348. 10.1038/nature00829 12124627

[B58] Liu H. Heaney A. P. (2011). Refined fructose and cancer. Expert Opin. Ther. Targets 15, 1049–1059. 10.1517/14728222.2011.588208 21623683

[B59] Matsuo K. Hiraki A. Ito H. Kosaka T. Suzuki T. Hirose K.. (2008). Soy consumption reduces the risk of non-small-cell lung cancers with epidermal growth factor receptor mutations among Japanese. Cancer Sci. 99, 1202–1208. 10.1111/j.1349-7006.2008.00812.x 18429954 PMC11159498

[B60] Mattison J. A. Wright C. Bronson R. T. Roth G. S. Ingram D. K. Bartke A. (2000). Studies of aging in ames dwarf mice: effects of caloric restriction. J. Am. Aging Assoc. 23, 9–16. 10.1007/s11357-000-0002-0 23604794 PMC3455356

[B61] Mattson M. P. Shea T. B. (2003). Folate and homocysteine metabolism in neural plasticity and neurodegenerative disorders. Trends Neurosci. 26, 137–146. 10.1016/S0166-2236(03)00032-8 12591216

[B62] Maxwell J. R. Gowers I. R. Moore D. J. Wilson A. G. (2010). Alcohol consumption is inversely associated with risk and severity of rheumatoid arthritis. Rheumatology 49, 2140–2146. 10.1093/rheumatology/keq202 20667949

[B63] Mayne S. T. Playdon M. C. Rock C. L. (2016). Diet, nutrition, and cancer: past, present and future. Nat. Rev. Clin. Oncol. 13, 504–515. 10.1038/nrclinonc.2016.24 26951041

[B64] Melmed S. (2011). Pathogenesis of pituitary tumors. Nat. Rev. Endocrinol. 7, 257–266. 10.1038/nrendo.2011.40 21423242

[B65] Menche J. Sharma A. Kitsak M. Ghiassian S. D. Vidal M. Loscalzo J.. (2015). Disease networks. Uncovering disease-disease relationships through the incomplete interactome. Science 347:1257601. 10.1126/science.1257601 25700523 PMC4435741

[B66] Miller J. A. Horvath S. Geschwind D. H. (2010). Divergence of human and mouse brain transcriptome highlights Alzheimer disease pathways. Proc. Natl. Acad. Sci. U.S.A. 107, 12698–12703. 10.1073/pnas.0914257107 20616000 PMC2906579

[B67] Mishra R. Bhadauria S. Murthy P. K. Murthy P. S. (2011). Glycine soya diet synergistically enhances the suppressive effect of tamoxifen and inhibits tamoxifen-promoted hepatocarcinogenesis in 7,12-dimethylbenz[alpha]anthracene-induced rat mammary tumor model. Food Chem. Toxicol. 49, 434–440. 10.1016/j.fct.2010.11.020 21092749

[B68] Moo-Huchin V. M. Moo-Huchin M. I. Estrada-Leon R. J. Cuevas-Glory L. Estrada-Mota I. A. Ortiz-Vazquez E.. (2015). Antioxidant compounds, antioxidant activity and phenolic content in peel from three tropical fruits from Yucatan, Mexico. Food Chem. 166, 17–22. 10.1016/j.foodchem.2014.05.127 25053022

[B69] Motohashi N. Shirataki Y. Kawase M. Tani S. Sakagami H. Satoh K.. (2002). Cancer prevention and therapy with kiwifruit in Chinese folklore medicine: a study of kiwifruit extracts. J. Ethnopharmacol. 81, 357–364. 10.1016/S0378-8741(02)00125-3 12127237

[B70] Nencioni A. Grunebach F. Patrone F. Ballestrero A. Brossart P. (2007). Proteasome inhibitors: antitumor effects and beyond. Leukemia 21, 30–36. 10.1038/sj.leu.2404444 17096016

[B71] Ni Y. Li J. Panagiotou G. (2015). A molecular-level landscape of diet-gut microbiome interactions: toward dietary interventions targeting bacterial genes. MBio 6:e01263-15. 10.1128/mBio.01263-15 26507230 PMC4626853

[B72] O'Keefe S. J. Li J. V. Lahti L. Ou J. Carbonero F. Mohammed K.. (2015). Fat, fibre and cancer risk in African Americans and rural Africans. Nat. Commun. 6:6342. 10.1038/ncomms7342 25919227 PMC4415091

[B73] Ono K. Han J. (2000). The p38 signal transduction pathway: activation and function. Cell. Signal. 12, 1–13. 10.1016/S0898-6568(99)00071-6 10676842

[B74] Pacini C. Iorio F. Goncalves E. Iskar M. Klabunde T. Bork P.. (2013). DvD: an R/Cytoscape pipeline for drug repurposing using public repositories of gene expression data. Bioinformatics 29, 132–134. 10.1093/bioinformatics/bts656 23129297 PMC3530913

[B75] Panagiotou G. Nielsen J. (2009). Nutritional systems biology: definitions and approaches. Annu. Rev. Nutr. 29, 329–339. 10.1146/annurev-nutr-080508-141138 19575602

[B76] Parkinson H. Sarkans U. Shojatalab M. Abeygunawardena N. Contrino S. Coulson R.. (2005). ArrayExpress–a public repository for microarray gene expression data at the EBI. Nucleic Acids Res. 33, D553–D555. 10.1093/nar/gki056 15608260 PMC540010

[B77] Pavlova N. N. Thompson C. B. (2016). The emerging hallmarks of cancer metabolism. Cell Metab. 23, 27–47. 10.1016/j.cmet.2015.12.006 26771115 PMC4715268

[B78] Pi-Sunyer X. (2005). Do glycemic index, glycemic load, and fiber play a role in insulin sensitivity, disposition index, and type 2 diabetes? Diabetes Care 28, 2978–2979. 10.2337/diacare.28.12.2978 16306566

[B79] Port A. M. Ruth M. R. Istfan N. W. (2012). Fructose consumption and cancer: is there a connection? Curr. Opin. Endocrinol. Diabetes Obes. 19, 367–374. 10.1097/MED.0b013e328357f0cb 22922366

[B80] Risbridger G. P. Davis I. D. Birrell S. N. Tilley W. D. (2010). Breast and prostate cancer: more similar than different. Nat. Rev. Cancer 10, 205–212. 10.1038/nrc2795 20147902

[B81] Ross Z. M. O'Gara E. A. Hill D. J. Sleightholme H. V. Maslin D. J. (2001). Antimicrobial properties of garlic oil against human enteric bacteria: evaluation of methodologies and comparisons with garlic oil sulfides and garlic powder. Appl. Environ. Microbiol. 67, 475–480. 10.1128/AEM.67.1.475-480.2001 11133485 PMC92605

[B82] Sayers E. W. Barrett T. Benson D. A. Bryant S. H. Canese K. Chetvernin V.. (2009). Database resources of the National Center for Biotechnology Information. Nucleic Acids Res. 37, D5–D15. 10.1093/nar/gkp382 18940862 PMC2686545

[B83] Schoonen W. M. Salinas C. A. Kiemeney L. A. Stanford J. L. (2005). Alcohol consumption and risk of prostate cancer in middle-aged men. Int. J. Cancer 113, 133–140. 10.1002/ijc.20528 15386436

[B84] Shigemizu D. Hu Z. Hung J. H. Huang C. L. Wang Y. DeLisi C. (2012). Using functional signatures to identify repositioned drugs for breast, myelogenous leukemia and prostate cancer. PLoS Comput. Biol. 8:e1002347. 10.1371/journal.pcbi.1002347 22346740 PMC3276504

[B85] Shu X. O. Jin F. Dai Q. Wen W. Potter J. D. Kushi L. H.. (2001). Soyfood intake during adolescence and subsequent risk of breast cancer among Chinese women. Cancer Epidemiol. Biomarkers Prev. 10, 483–488. 11352858

[B86] Sikkema M. de Jonge P. J. Steyerberg E. W. Kuipers E. J. (2010). Risk of esophageal adenocarcinoma and mortality in patients with Barrett's esophagus: a systematic review and meta-analysis. Clin. Gastroenterol. Hepatol. 8, 235–244; quiz e232. 10.1016/j.cgh.2009.10.010 19850156

[B87] Sirota M. Dudley J. T. Kim J. Chiang A. P. Morgan A. A. Sweet-Cordero A.. (2011). Discovery and preclinical validation of drug indications using compendia of public gene expression data. Sci. Transl. Med. 3:96ra77. 10.1126/scitranslmed.3001318 21849665 PMC3502016

[B88] Solon-Biet S. M. McMahon A. C. Ballard J. W. Ruohonen K. Wu L. E. Cogger V. C.. (2014). The ratio of macronutrients, not caloric intake, dictates cardiometabolic health, aging, and longevity in *ad libitum*-fed mice. Cell Metab. 19, 418–430. 10.1016/j.cmet.2014.02.009 24606899 PMC5087279

[B89] Sotiriou C. Pusztai L. (2009). Gene-expression signatures in breast cancer. N. Engl. J. Med. 360, 790–800. 10.1056/NEJMra0801289 19228622

[B90] Speakman J. R. Mitchell S. E. (2011). Caloric restriction. Mol. Aspects Med. 32, 159–221. 10.1016/j.mam.2011.07.001 21840335

[B91] Subramanian A. Tamayo P. Mootha V. K. Mukherjee S. Ebert B. L. Gillette M. A.. (2005). Gene set enrichment analysis: a knowledge-based approach for interpreting genome-wide expression profiles. Proc. Natl. Acad. Sci. U.S.A. 102, 15545–15550. 10.1073/pnas.0506580102 16199517 PMC1239896

[B92] Suzuki R. Shimodaira H. (2006). Pvclust: an R package for assessing the uncertainty in hierarchical clustering. Bioinformatics 22, 1540–1542. 10.1093/bioinformatics/btl117 16595560

[B93] Valko M. Leibfritz D. Moncol J. Cronin M. T. Mazur M. Telser J. (2007). Free radicals and antioxidants in normal physiological functions and human disease. Int. J. Biochem. Cell Biol. 39, 44–84. 10.1016/j.biocel.2006.07.001 16978905

[B94] van Noort V. Scholch S. Iskar M. Zeller G. Ostertag K. Schweitzer C.. (2014). Novel drug candidates for the treatment of metastatic colorectal cancer through global inverse gene-expression profiling. Cancer Res. 74, 5690–5699. 10.1158/0008-5472.CAN-13-3540 25038229

[B95] Vinha A. F. Alves R. C. Barreira S. V. Costa A. S. Oliveira M. B. (2015). Impact of boiling on phytochemicals and antioxidant activity of green vegetables consumed in the Mediterranean diet. Food Funct. 6, 1157–1163. 10.1039/C4FO01209G 25690509

[B96] Wang X. (2009). The antiapoptotic activity of melatonin in neurodegenerative diseases. CNS Neurosci. Ther. 15, 345–357. 10.1111/j.1755-5949.2009.00105.x 19818070 PMC2846661

[B97] Westergaard D. Li J. Jensen K. Kouskoumvekaki I. Panagiotou G. (2014). Exploring mechanisms of diet-colon cancer associations through candidate molecular interaction networks. BMC Genomics 15:380. 10.1186/1471-2164-15-380 24886433 PMC4055784

[B98] Wiseman M. (2008). The second World Cancer Research Fund/American Institute for Cancer Research expert report. Food, nutrition, physical activity, and the prevention of cancer: a global perspective. Proc. Nutr. Soc. 67, 253–256. 10.1017/S002966510800712X 18452640

[B99] Xie Y. Qin J. Nan G. Huang S. Wang Z. Su Y. (2016). Coffee consumption and the risk of lung cancer: an updated meta-analysis of epidemiological studies. Eur. J. Clin. Nutr. 70, 199–206. 10.1038/ejcn.2015.96 26081490

[B100] Yang X. Sun X. (2007). Meta-analysis of several gene lists for distinct types of cancer: a simple way to reveal common prognostic markers. BMC Bioinformatics 8:118. 10.1186/1471-2105-8-118 17411443 PMC1853113

[B101] Yeoh E. J. Ross M. E. Shurtleff S. A. Williams W. K. Patel D. Mahfouz R.. (2002). Classification, subtype discovery, and prediction of outcome in pediatric acute lymphoblastic leukemia by gene expression profiling. Cancer Cell 1, 133–143. 10.1016/S1535-6108(02)00032-6 12086872

[B102] Zhang M. Xie X. Lee A. H. Binns C. W. (2004). Soy and isoflavone intake are associated with reduced risk of ovarian cancer in southeast china. Nutr. Cancer 49, 125–130. 10.1207/s15327914nc4902_2 15489204

[B103] Zhang M. Yang Z. Y. Binns C. W. Lee A. H. (2002). Diet and ovarian cancer risk: a case-control study in China. Br. J. Cancer 86, 712–717. 10.1038/sj.bjc.6600085 11875731 PMC2375319

[B104] Zheng-Bradley X. Rung J. Parkinson H. Brazma A. (2010). Large scale comparison of global gene expression patterns in human and mouse. Genome Biol. 11:R124. 10.1186/gb-2010-11-12-r124 21182765 PMC3046484

