# Supplementary material for: Designing Dietary Recommendations Using System Level Interactomics Analysis and Network-Based Inference
Source: Front Physiol. 2017 Sep 28;8:753. doi: 10.3389/fphys.2017.00753 (PMC5625024; doi:10.3389/fphys.2017.00753)
Supplement: Supplementary file 9 [file Image1.pdf]

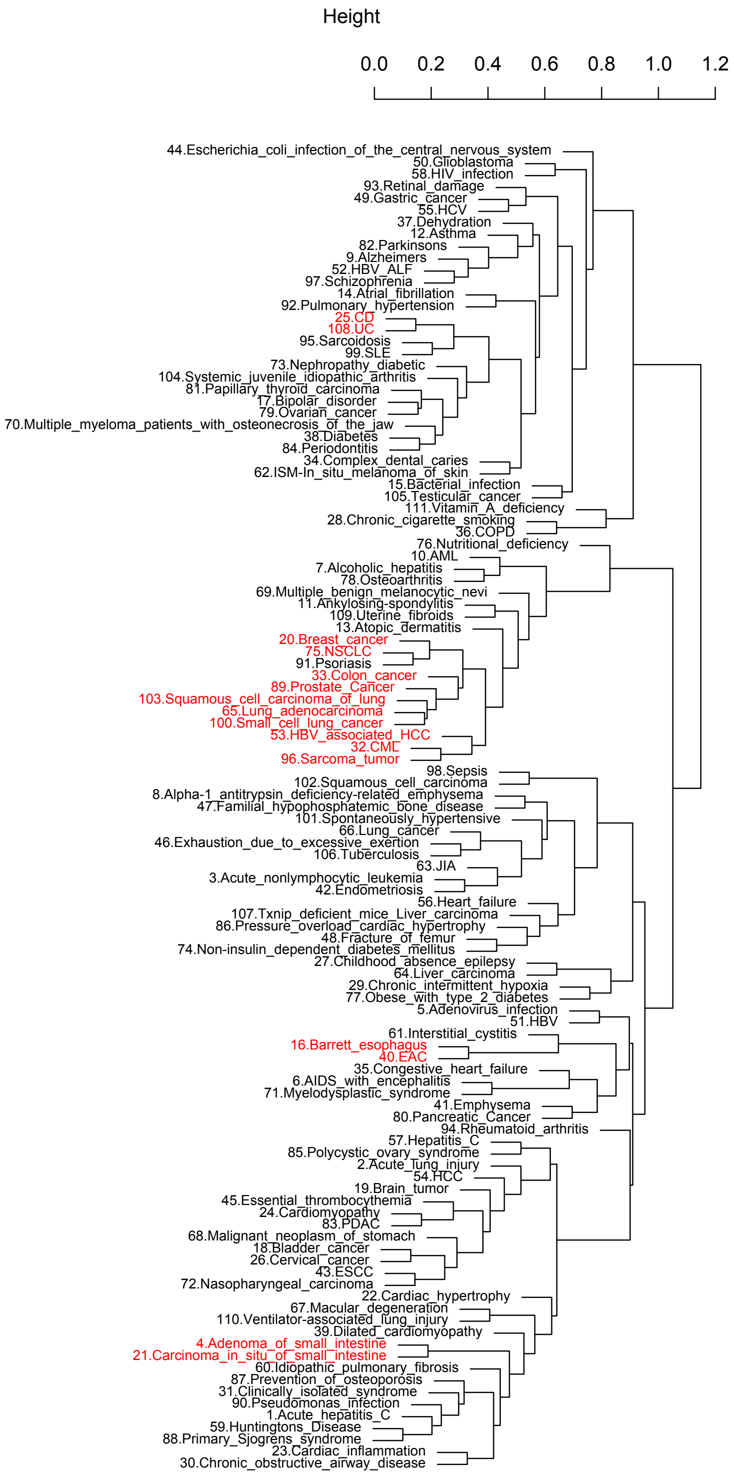

Figure S1. Hierarchical clustering of diseases by drug-disease enrichment scores. The number before each disease name was used as the disease ID. Diseases are clustered based on the enrichment scores of diseases across all drugs. The clusters highlighted with red are known to share characteristic pathophysiology.
