# Supplementary material for: Designing Dietary Recommendations Using System Level Interactomics Analysis and Network-Based Inference
Source: Front Physiol. 2017 Sep 28;8:753. doi: 10.3389/fphys.2017.00753 (PMC5625024; doi:10.3389/fphys.2017.00753)
Supplement: Supplementary file 10 [file Image2.pdf]

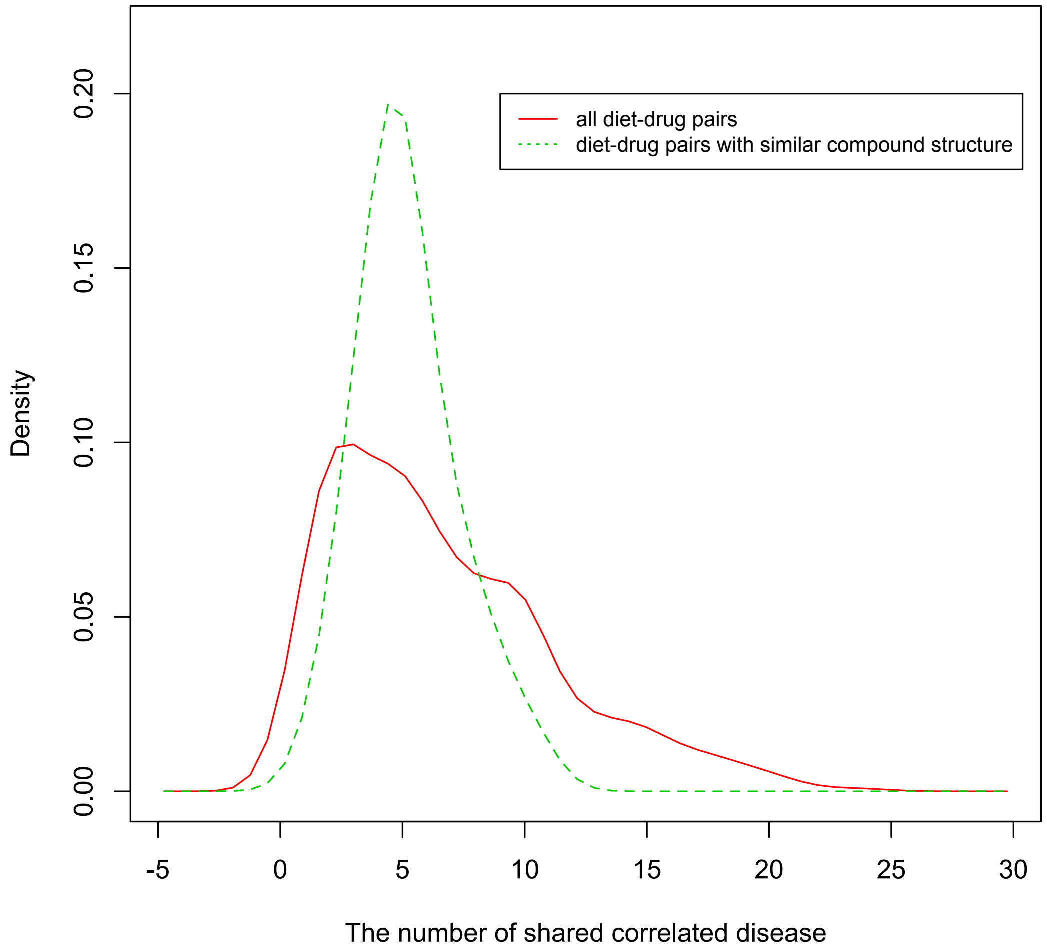

Fig S2. Density plot showing the distribution of the number of anti-correlated diseases shared by diet-drug pairs. Diet-drug pairs with similar compound structures tend to share more anti-correlated diseases.
