# Supplementary material for: Designing Dietary Recommendations Using System Level Interactomics Analysis and Network-Based Inference
Source: Front Physiol. 2017 Sep 28;8:753. doi: 10.3389/fphys.2017.00753 (PMC5625024; doi:10.3389/fphys.2017.00753)
Supplement: Supplementary file 11 [file Image3.pdf]

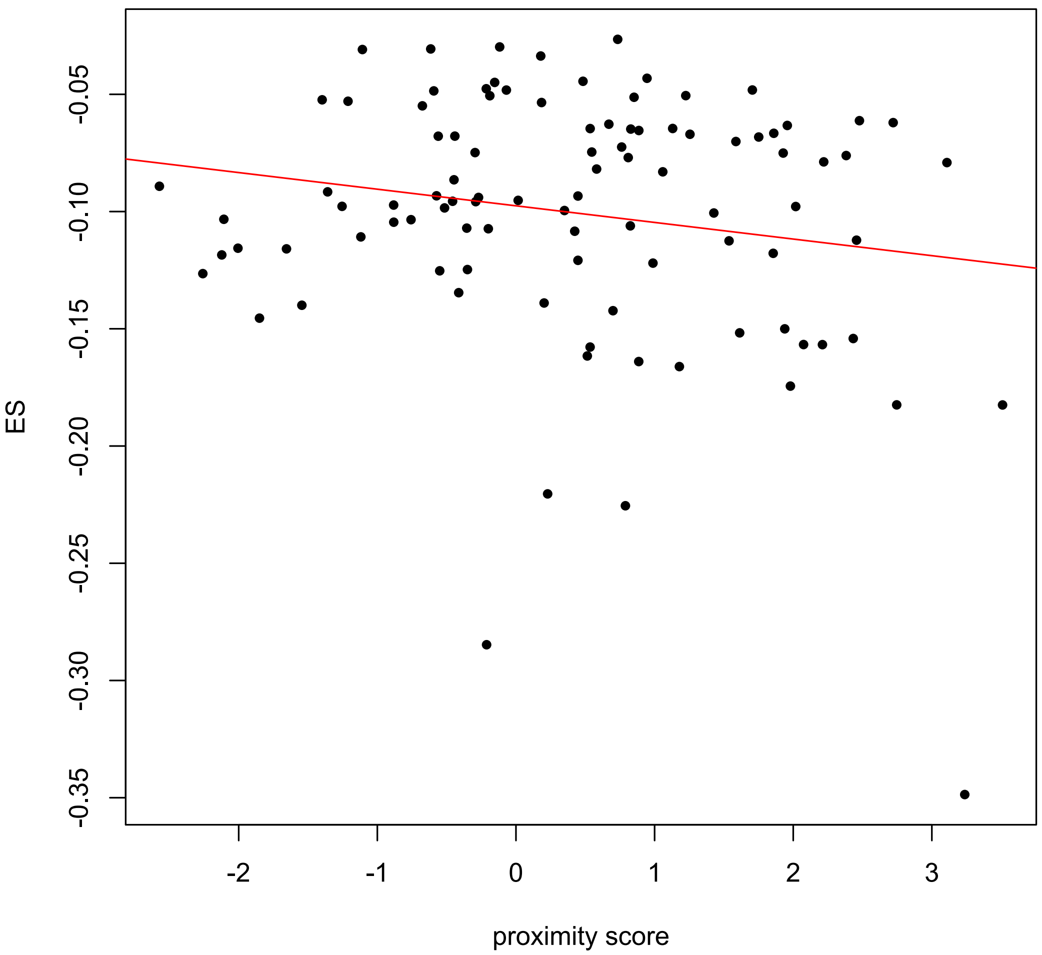

Fig S3. Scatter plot showing the relationship between enrichment score and proximity score. No significant correlation was observed between ES and proximity score (Spearman's correlation coefficient = -0.08, P = 0.407)
