# Supplementary figures and images for: Designing Dietary Recommendations Using System Level Interactomics Analysis and Network-Based Inference
Source: Front Physiol. 2017 Sep 28;8:753. doi: 10.3389/fphys.2017.00753 (PMC5625024; doi:10.3389/fphys.2017.00753)

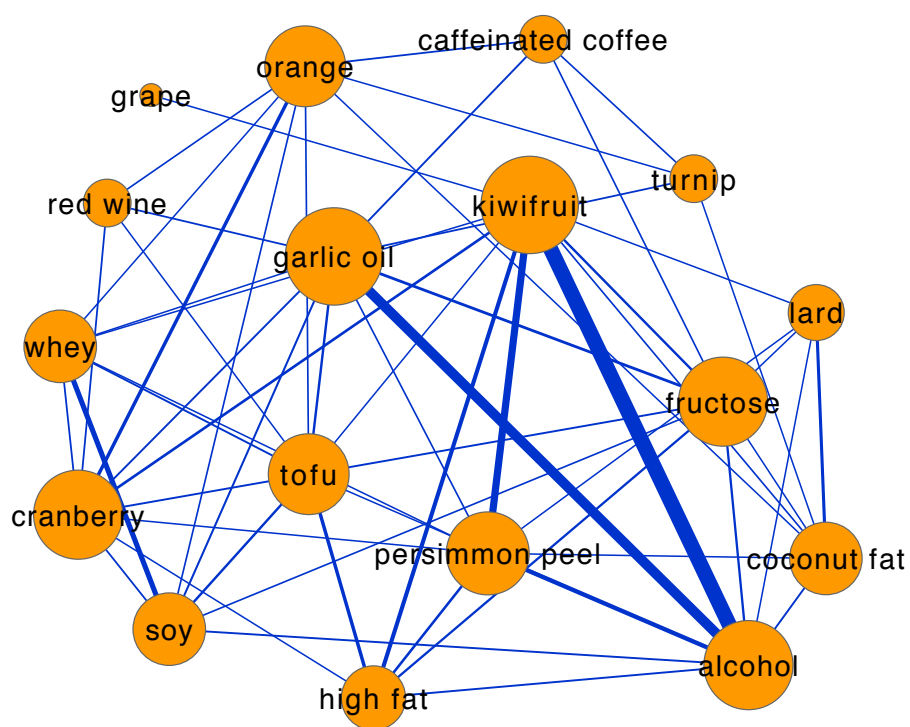

**Fig S4. Synergistic food pairs network.**

Supplement: Supplementary file 12 [file Image4.pdf]
