# Supplementary material for: Designing Dietary Recommendations Using System Level Interactomics Analysis and Network-Based Inference
Source: Front Physiol. 2017 Sep 28;8:753. doi: 10.3389/fphys.2017.00753 (PMC5625024; doi:10.3389/fphys.2017.00753)
Supplement: Supplementary file 13 [file Image5.pdf]

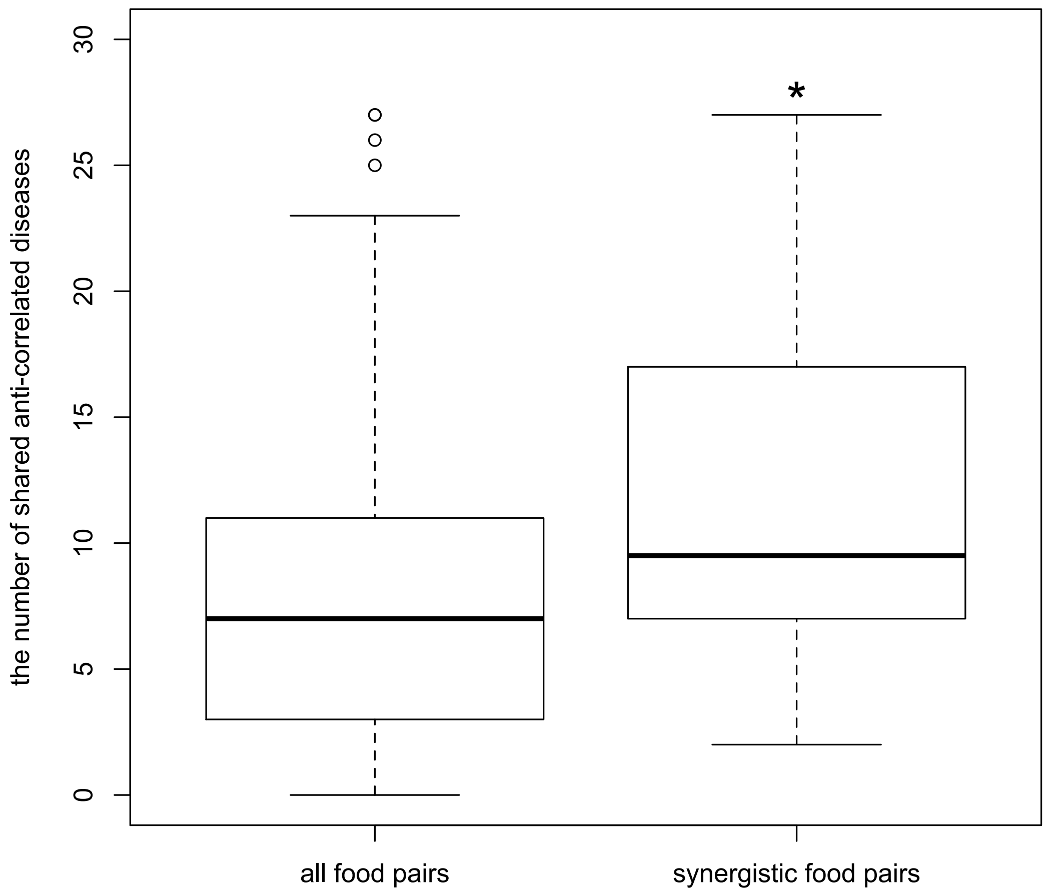

Fig S5. Food pairs sharing anti-correlated diseases. The plot shows synergistic food pairs that we identified as tending to share more anti-correlated diseases (Wilcoxon ranked sum test, P = 0.00018).
